# Identification of patients’ smoking status using an explainable AI approach: a Danish electronic health records case study

**DOI:** 10.1186/s12874-024-02231-4

**Published:** 2024-05-17

**Authors:** Ali Ebrahimi, Margrethe Bang Høstgaard Henriksen, Claus Lohman Brasen, Ole Hilberg, Torben Frøstrup Hansen, Lars Henrik Jensen, Abdolrahman Peimankar, Uffe Kock Wiil

**Affiliations:** 1 SDU Health Informatics and Technology, The Maersk Mc-Kinney Moller Institute, University of Southern Denmark, Odense, 5230 Denmark; 2 Department of Oncology, Lillebaelt Hospital, University Hospital of Southern Denmark, Vejle, 7100 Denmark; 3 Department of Biochemistry and Immunology, Lillebaelt Hospital, University Hospital of Southern Denmark, Vejle, 7100 Denmark; 4 Institute of Regional Health Research, University of Southern Denmark, Odense, Denmark; 5 Department of Internal Medicine, Lillebaelt Hospital, University Hospital of Southern Denmark, Vejle, 7100 Denmark

**Keywords:** Natural language processing, Text classification, Stacking-based ensemble, Deep learning, CNN, LSTM, Explainable Artificial Intelligence (XAI), Electronic health record, Smoking status

## Abstract

**Background:**

Smoking is a critical risk factor responsible for over eight million annual deaths worldwide. It is essential to obtain information on smoking habits to advance research and implement preventive measures such as screening of high-risk individuals. In most countries, including Denmark, smoking habits are not systematically recorded and at best documented within unstructured free-text segments of electronic health records (EHRs). This would require researchers and clinicians to manually navigate through extensive amounts of unstructured data, which is one of the main reasons that smoking habits are rarely integrated into larger studies. Our aim is to develop machine learning models to classify patients’ smoking status from their EHRs.

**Methods:**

This study proposes an efficient natural language processing (NLP) pipeline capable of classifying patients’ smoking status and providing explanations for the decisions. The proposed NLP pipeline comprises four distinct components, which are; (1) considering preprocessing techniques to address abbreviations, punctuation, and other textual irregularities, (2) four cutting-edge feature extraction techniques, i.e. Embedding, BERT, Word2Vec, and Count Vectorizer, employed to extract the optimal features, (3) utilization of a Stacking-based Ensemble (SE) model and a Convolutional Long Short-Term Memory Neural Network (CNN-LSTM) for the identification of smoking status, and (4) application of a local interpretable model-agnostic explanation to explain the decisions rendered by the detection models. The EHRs of 23,132 patients with suspected lung cancer were collected from the Region of Southern Denmark during the period 1/1/2009-31/12/2018. A medical professional annotated the data into ‘Smoker’ and ‘Non-Smoker’ with further classifications as ‘Active-Smoker’, ‘Former-Smoker’, and ‘Never-Smoker’. Subsequently, the annotated dataset was used for the development of binary and multiclass classification models. An extensive comparison was conducted of the detection performance across various model architectures.

**Results:**

The results of experimental validation confirm the consistency among the models. However, for binary classification, BERT method with CNN-LSTM architecture outperformed other models by achieving precision, recall, and F1-scores between 97% and 99% for both Never-Smokers and Active-Smokers. In multiclass classification, the Embedding technique with CNN-LSTM architecture yielded the most favorable results in class-specific evaluations, with equal performance measures of 97% for Never-Smoker and measures in the range of 86 to 89% for Active-Smoker and 91–92% for Never-Smoker.

**Conclusion:**

Our proposed NLP pipeline achieved a high level of classification performance. In addition, we presented the explanation of the decision made by the best performing detection model. Future work will expand the model’s capabilities to analyze longer notes and a broader range of categories to maximize its utility in further research and screening applications.

**Supplementary Information:**

The online version contains supplementary material available at 10.1186/s12874-024-02231-4.

## Introduction

Information on smoking status is crucial especially in cardiovascular, pulmonary, diabetes, and cancer research, since in addition to being a common risk factor it is also a confounder for various diseases [1]. Smoking accounts for more than eight million deaths annually [2]. In the specific area of lung cancer, the implementation of screening and detective models is becoming more relevant. The models, however, lack the ability to identify high-risk individuals who are dependent on tobacco [3]. In Denmark, smoking habits are not formally registered unless patients are diagnosed with cancer or a chronic disease that includes them in the National Clinical Registries. For example, information on the smoking habits of a lung cancer patient will be registered in the Danish Lung Cancer Registry, while a patient with chronic obstructive pulmonary disease, followed at a hospital level, will appear in the Danish Register of Chronic Obstructive Lung Disease [4]. Patients with milder conditions often do not appear in national registries, and information on smoking habits is only available as unstructured free-text in electronic health records (EHRs) [5]. The records often have an unrestricted format leading to differences between clinicians in terms of spelling errors, abbreviations, and a field-specific jargon that may be difficult for outsiders to interpret [6]. Clinicians have to manually search for smoking habits, which is feasible when dealing with a small number of patients, but it becomes impractical with larger cohorts, such as a high-risk population for lung cancer screening or large-scale research with smoking as an essential risk factor [7].

Natural Language Processing (NLP), a sub-field of artificial intelligence, focuses on analyzing linguistic data, particularly unstructured textual data using machine learning. The main goal of NLP is to transform free text into structured data that can be easily identified by machines [8]. NLP has been used in healthcare for various tasks such as detecting heart failure criteria [9], identifying adverse drug effects [10] detecting symptoms of specific disease, and improving quality of life [11]. In 2006, the “Informatics for Integrating Biology and the Bedside” research center announced the “Smoking challenge” funded by the National Institute of Health in the USA. The challenge aimed to address the problem of classifying smoking status based on EHRs and compare the performance with classifications made by pulmonologists. By means of supervised and unsupervised classifiers, several models demonstrated the ability to classify smoking status using a limited number of key textual features [12]. More recently, applying deep neural networks to EHRs has been in focus due to their better performance and lower preprocessing requirements [13, 14]. In 2018, Google developed a new technique called Bidirectional Encoder Representations from Transformers (BERT). Unlike traditional word embedding methods such as word2vec, BERT is context-sensitive and generates a representation of each word based on the other words in the sentence [15, 16]. BERT is considered state-of-the-art, as it allows for transfer learning and adaptations to other domains [17]. In 2020, a Danish edition of BERT was introduced, trained on 1.6 billion words from various online repositories (Common Crawl, Wikipedia, OpenSubtitles, etc.) [18, 19]. Additionally, in 2021, Derczynski et al. presented the first Danish Gigaword Corpus, a billion-word corpus encompassing a wide range of the Danish language from different domains, settings, time periods, registers, and dialects [20].

Despite the advancements, a high-performing model capable of detection smoking status in the Danish language is yet to be developed. This limitation can be attributed to both the limited availability of text data due to access restrictions and the lack of advanced model development. The complex structure of EHRs further limits the possibility of transfer learning from other languages [21]. Consequently, this paper aims to address these challenges by presenting a high-performing NLP-based model capable of detecting smoking status in Danish EHRs using both binary and multiclass labels. The model is expected to be valuable in future screening scenarios and various research fields, including other types of cancer and cardiovascular diseases.

As machine learning and deep neural networks continue to advance, they often remain mysterious for both developers and end-users, resembling black boxes. The lack of transparency obstructs the broad adoption of such models, especially in domains where decision making holds critical importance such as the medical field. To effectively implement a model in a medical context, explainability becomes imperative in allowing clinicians and researchers to trust and comprehend the precise detection made [22]. An explainable model enhances the chance of identifying systematic errors and hence improves the model’s performance. Understanding the rationale behind a detection and the potential for model enhancement is of utmost importance for clinicians or researchers who will ultimately be responsible for the outcomes. While research and applications in explainable artificial intelligence have grown in the context of image and structured data models, those based on free-text datasets have received comparatively less attention [23]. Consequently, in addition to developing highly accurate detection models, this study seeks to provide transparent post-hoc explanations for the models.

The contributions of our study can be summarized as follows:

1. *Formulate several NLP-based architectures to identify smoking status*: To the best of our knowledge, this is the first study to detect smoking status based on Danish text from EHR. Several NLP-based architectures formulated resulting from the integration of advanced feature extraction techniques with ensemble-based machine learning and deep learning models.
2. *Analyzing the detection performance of developed architectures and comparing them with state-of-the-art detection models*: Comprehensive analysis and comparison of the performance of the developed models against existing state-of-the-art predictive models, with the superior models identified through rigorous statistical evaluation. This not only highlighted the detection performance of our model in comparison to others but also explored into a non-parametric statistical assessment based on the Friedman test.
3. *Post-hoc explanations for the detection models*: The study is the first study to provide model explanations for smoking status detection based on EHR. Explanation of the models’ decision-making processes using the state-of-the-art XAI approach, LIME, highlighting the significance of individual features and the underlying rationale for model decisions.

## Methods

**Fig. 1:**
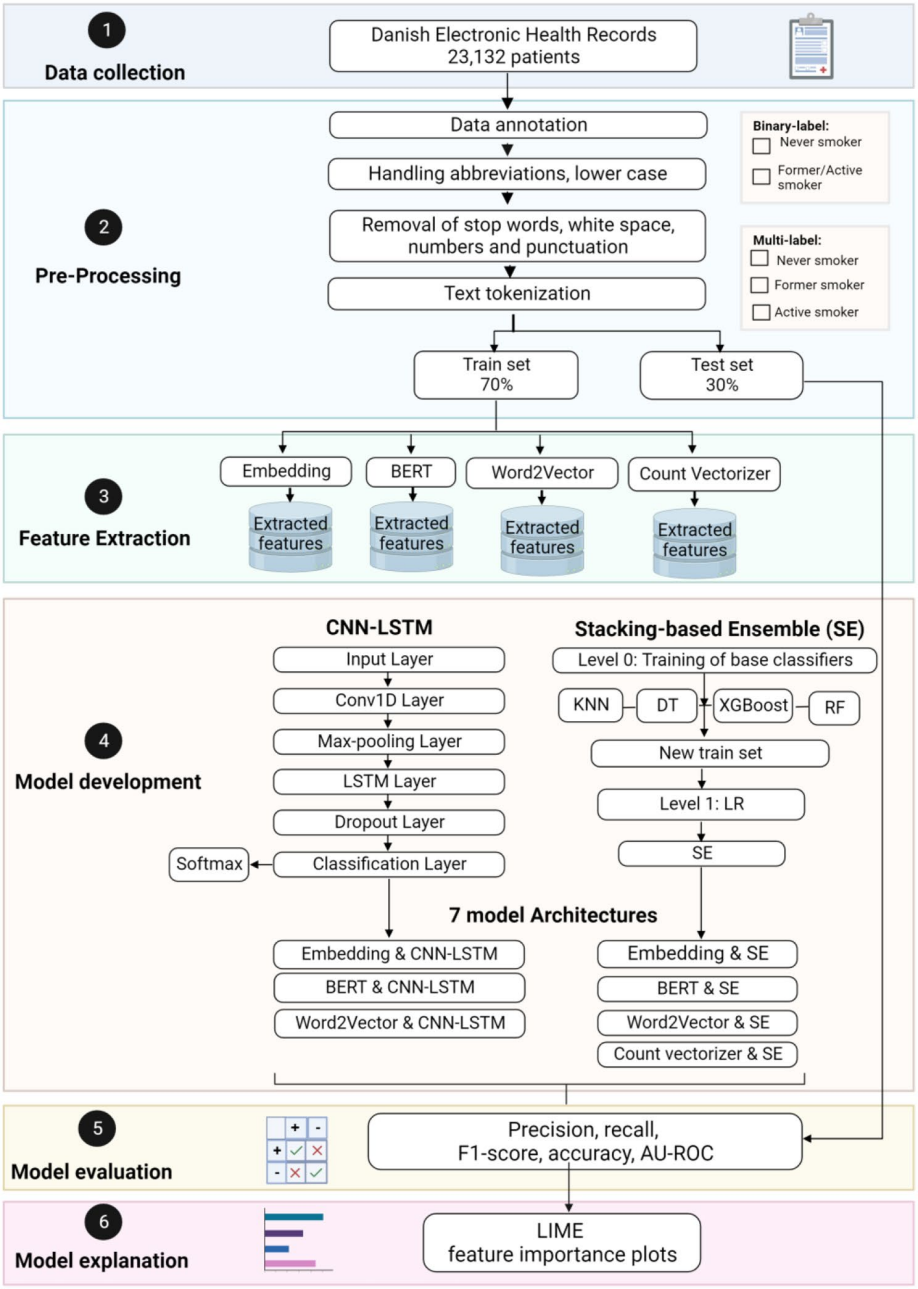
Flowchart depicting the study design in each step of the NLP pipeline. Bidirectional Encoder Representations from Transformers (BERT). Convolutional neural network with a long short-term memory layer (CNN-LSTM). K-Nearest Neighbors (KNN). Decision Tree. Created with Biorender.com

The subsequent sections outline the process of data collection followed by preprocessing, feature extraction, model development, evaluation, and explanation. Figure 1 provides a comprehensive overview of the study’s methodology, encompassing all stages of the pipeline.

### Data collection

Data for this project were obtained from EHRs within a cohort of 38,944 patients who underwent assessments for a potential risk of lung cancer between January 1, 2009 and December 31, 2018 in the Region of Southern Denmark. This cohort has been comprehensively described in a related work [24]. We collected all types of documents from the EHRs containing the subheaders “smoking” or “risk factors” without imposing any time constraints. The subheaders were chosen, as they most commonly contain documentation of smoking history. Moreover, the data annotation process would have been impractical which we used on complete patient notes from the EHRs. We carefully eliminated duplicate entries, instances with missing gender information, and we pseudonymized the data to ensure privacy and confidentiality.

### Pre-processing

Clinical notes underwent manual annotation by a medical doctor and the results were subsequently reviewed by the same doctor. The dataset underwent further refinement with a decision to include only one note per patient. As the annotated data have been employed in previous studies to predict lung cancer status, our selection focused on the note that provided the most comprehensive details regarding smoking status. Patients were primarily categorized as Active Smoker if they had detailed information on current pack-years (a widely recognized measure of smoking intensity calculated by multiplying the number of packs of cigarettes smoked per day by the number of years of smoking) [25]. The remaining patients, lacking information on pack-year, were categorized as Active-Smoker or Former-Smoker, or status unknown. To resolve the “unknown” category, additional notes for these patients were evaluated and a smoking label was assigned based on the note containing the most comprehensive information. Any duplicate entries were removed, retaining only the note responsible for the patient’s label.

To validate the smoking status annotation from the EHRs, the distributions were compared with registrations of pack-years obtained from the Danish Lung Cancer Registry. They had been recorded independently of the EHRs and manually completed by clinicians upon a patient’s lung cancer diagnosis. While the registration of smoking status from the Danish Lung Cancer Registry is not integrated in the EHRs, it is expected to align with the EHRs annotations overall. Following the annotation process the text underwent cleaning in the following sequence: Handling of abbreviations, conversion of all text to lowercase, removal of stop words, numbers, and punctuation marks. Consecutive spaces where there were two or more spaces in a row were either removed or converted to a single space. Finally, a word tokenizer was applied to convert sentences into word tokens.

The cleaning steps employed in this study are carefully tailored to enhance the analysis of Danish EHRs, recognizing the unique linguistic and structural characteristics of the language. Handling abbreviations initially is crucial in Danish language, where abbreviations can carry significant meanings or denote specific terminology, ensuring that such condensed forms are correctly interpreted or expanded for analysis. Converting all text to lowercase addresses the case sensitivity of Danish language, promoting uniformity and reducing the risk of duplicate representations for the same words.

The removal of stop words, numbers, and punctuation marks, beside the consolidation of consecutive spaces, streamlines the text, focusing the analysis on the most meaningful content without the noise of non-informative elements. This step is particularly effective in Danish, where functional words and punctuation can obscure key linguistic patterns if not properly managed. Applying a word tokenizer as the final step effectively breaks down sentences into individual tokens, a process that is essential for capturing the morphological richness of Danish words and phrases. Each of these steps, collectively, prepares the Danish EHRs for a more accurate and efficient computational analysis, ensuring that subsequent NLP tasks, such as feature extraction and model training, are performed on clean, consistent data that accurately reflects the intricacies of the Danish language.

Given the challenge of imbalanced class distribution, a stratified split approach was chosen, which entailed dividing the data into a training set (70%) and a test set (30%). By using a stratified split, the proportion of records in all classes remained consistent between the training and test sets. Preprocessing techniques, including data cleaning and feature extraction, were exclusively learned from the training set, and subsequently applied to the test set with necessary adaptations. This prevented a possible information leakage from the test set to the model training process, which could have led to an overly optimistic evaluation of model performance. It is important to note that the test set was exclusively used for evaluating the performance of the final models and did not contribute to the model learning process.

### Feature extraction

Before choosing a classification algorithm for the task, it is essential to transform the unstructured data into a numerically vectorized representation. Feature extraction can be done with word embedding methods referring to the representation of words and whole sentences in a numerical manner. Words are converted into numeric vectors, and vectors of words closely related would be closer to each other [26]. In this study, we consider three methods to encode the tokens of a given technical text into a vectorized representation: The well-known Word embedding, BERT, Count Vectorizer and Word2Vector. General descriptions of all methods are described in detail in Table 1. We applied a hyperparameter tuning step for the Count Vectorizer method using a randomized search cross validation to identify the threshold for the removal of frequent tokens and the number of n-grams.

**Table 1:** The four word embedding methods applied in the study

Feature extractor	Description
Embedding [27]	Embeddings transform text into dense vectors encapsulating semantic meanings in a compact space. These representations, often derived from neural networks trained on vast text datasets, ensure that similar words have closely aligned vectors. During training, the model adjusts vectors based on word context deepening the semantic understanding over time. The embeddings can represent entire sentences by techniques like averaging word vectors. The vectors can also be utilized as features in traditional machine learning models, bridging neural-based and classic techniques.
BERT [15]	BERT (Bidirectional Encoder Representations from Transformers) is a deep learning model designed for natural language understanding. When it comes to feature extraction, BERT captures contextual information from both directions (left-to-right and right-to-left) of a given word or token in a text. Instead of using the final outputs of BERT as predictions, one can extract the hidden states (vectors) from its layers as dense and context-rich representations of the text. Typically, the final hidden state (from the last layer) of the [CLS] token is used as a representation for the entire sequence, or individual token embeddings from desired layers can be averaged or pooled for sentence representations. The extracted features can then serve as input for other downstream tasks or traditional machine learning models, providing them with a rich, context-aware representation of the input text.
Count Vectorizer [28]	The count vectorizer is a technique used to extract words from a given text. In this process, the words are regarded as distinct features, and their classification is based on their frequency of occurrence. The information is represented as a vector, where each element corresponds to the frequency of a specific word within the document. By applying this method to each document, feature vectors are generated. The feature vectors capture the distribution of word frequencies across the entire corpus. Ultimately, the vectors are combined into a sparse matrix, in which each column represents a feature vector, thereby facilitating further analysis and modeling tasks.
Word2Vector [29]	Word2Vector is a word embedding method for generating n-dimensional vectors from words. A word2vec model is trained on a corpus of text data whereby it learns the relationships between words as they occur in the corpus through the use of a neural network. This network can then be used to convert words to vectors. The relationship of words is encoded in this embedded space through the relative position of individual words in the space. The resulting embedding is similar to that produced by transformers, but unlike transformers, the vectors produced by the word2vector algorithm do not contain any information on the context of the word as it appears in a specific sentence but merely an amalgamation of all contexts the given word has appeared in.

Selecting Word Embedding, BERT, Count Vectorizer, and Word2Vec as methods for encoding tokens of Danish EHR into vector representations aligns with our objective to capture the linguistic nuances inherent to the Danish language effectively. Word Embeddings and Word2Vec, both deeply rooted in learning contextual relationships and semantic similarities, are particularly adept at navigating the intricate morphological characteristics of Danish language, such as its compound words and diverse verb forms. These methods excel in creating nuanced vector representations that reflect the semantic richness of words within their specific context, a crucial feature for the Danish language with its nuanced meanings and expressions.

BERT, with its deep contextualized training, excels in understanding the syntax and semantics of Danish text, leveraging its transformer architecture to capture subtle language cues and idiomatic expressions unique to Danish language. This is particularly beneficial given the contextual richness and syntactic flexibility of Danish. Lastly, Count Vectorizer provides a straightforward yet powerful approach to text representation, capturing the frequency of terms in a manner that supports the identification of domain-specific terminology prevalent in technical texts. Additionally, these methods provide a comprehensive toolkit for Danish text analysis, balancing deep semantic understanding with robust statistical approaches to ensure accurate and meaningful representation of Danish EHR.

### Model development

#### Stacking-based ensemble (SE)

The SE method was created by Wolpert et al. and is different from previous ensemble learning techniques in that it employs meta-learning to combine multiple types of machine learning algorithms [30]. SE is used in a two-level structure where the level-1 meta learner combines the outputs of the level-0 base learners. Figure 1, Sect. 4 illustrates the stacking structure used in this study, which comprises three stages. The first stage involves training the base classifiers, which are K-Nearest Neighbor, Decision Trees, Random Forest, and XGBoost algorithms. The second stage involves gathering the output detection (feature vectors) of the base classifiers to generate a new reorganized training set. Finally, in the third stage, the Logistic Regression algorithm is utilized to train the meta-classifier using the new training set, resulting in the development of SE. Detailed descriptions of the developed machine learning algorithms are provided in Table 2.

**Table 2:** Description of machine learning algorithms used and the stacking-based ensemble method (SE). K-Nearest Neighbors (KNN). Decision Tree (DT). Extreme Gradient Boosting (XG-Boost). Random Forest (RF).

Model	Description
KNN [31]	K-Nearest Neighbor (KNN) is a classifier in the category of non-parametric methods. Its approach to classifying an unknown instance involves examining the classification of its neighboring data points. Specifically, it labels the target by evaluating the class labels of k number of the closest points in the feature space. The classification of the target is then determined by assigning it to the most frequently occurring class among its k-nearest neighbors.
DT [32]	The Decision Tree (DT) algorithm is a recursive and greedy approach that uses a tree data structure where nodes and branches represent targets and features, respectively. The initial node in the tree is the root node from which other nodes branch out. The algorithm uses all nodes, including the leaves, to determine the best class for the target. The DT algorithm constructs the tree by first growing it to its maximum depth to ensure that each leaf node is pure. It then performs a pruning upwards, optimizing the classification error as well as the proportion of final nodes in the tree.
RF [33]	Random Forest (RF) is a commonly used bagging ensemble algorithm in health-related research. Essentially, RF is a group of classifiers made up of decision trees generated from two different sources of randomization. Firstly, a random sample is trained on each decision tree with the original data replaced by new information on the same size as the supplied training set. It is estimated that the resulting bootstrapping process includes approximately 37% redundant instances.
XGBoost [34]	XGBoost is an ensemble method that employs decision trees and utilizes the gradient boosting framework. It is renowned for its versatility, portability, and efficiency. Unlike traditional gradient boosting, XGBoost approximates the optimization of the objective function using the second-order derivative (or Taylor expansion). It offers a variety of hyperparameters that give practitioners a fine-tuned control over model training. Initially, the algorithm makes a naive prediction for the target variable. To enhance the prediction’s accuracy, XGBoost iteratively constructs new trees that focus on the residuals or errors of the preceding trees. When each tree has been trained, its contribution to the final prediction is moderated by a learning rate, preventing overfitting and ensuring more robust generalization.
LR [35]	Logistic Regression is a statistical method used for modeling the probability of a binary outcome based on one or more predictor variables. The logistic function transforms any linear combination of the predictors into a value between 0 and 1 suitable for estimating probabilities, which can then be translated into class predictions. Due to its interpretability and simplicity, Logistic Regression is commonly employed in various fields, including medicine, where it is used to relate patient characteristics to outcomes.
SE [36]	The stacking method is a popular type of heterogeneous ensemble learning that uses meta-models to combine various base classifiers to generate more accurate predictions. The primary advantage of the stacking method is that it can leverage the strengths of multiple effective models to produce more precise forecasts. The stacking method is trained on the complete training set, and a meta estimator is used to learn how to combine the base classifiers, which is distinct from other ensemble learning techniques such as Random Forest. The stacking method can assess the error of all base classifiers separately using basic learning processes and subsequently reduce residual errors using meta-learning steps.

#### CNN-LSTM

For the detection of smoking status, we also used the architecture CNN-LSTM. It consists of five layers, i.e., an input layer for word embedding, a one-dimensional convolutional network layer for local feature extraction, an LSTM network layer for capturing long-term dependencies, a dropout layer, and a classification layer for label detection. The structure of our model is shown in Fig. 1 (Sect. 4). In the input layer, input texts are treated as a matrix. Each row of the matrix represents a word, derived from the feature extraction method. In this study, the dimension of 300 is considered for the input layer. We used a one-dimensional convolution layer (Conv1D) to capture the sequence information and reduce the dimensions of the input data. A convolution operation involves a convolutional kernel applied to a fixed window of words to compute a new feature. The kernel, also called a filter, completes the feature extraction. Each filter is applied to a window of m words to obtain a single feature. To ensure the integrity of the word as the smallest granularity, the width of the filter is equal to the width of the original matrix. In this study, we employed the Conv1D layer with 256 filters and a kernel size of 3 in the output of the embedding layer to learn the lower-level features from words. A nonlinear activation function ReLU is used to reduce the number of iterations needed for convergence in deep networks.

Following the above steps, the result of the convolution was pooled using the maximum pooling operation to capture essential features in the text. To improve the quality of our text classification task, the different calculated features were concatenated to constitute the input of the LSTM layer. LSTM solves the vanishing gradient problem because it learns to regulate the flow of information. Due to high memory power, LSTMs can efficiently capture contextual information from the input text and produce high-level features that are used for further classification. We added a dropout layer to reduce the chance of overfitting. Finally, the last component is the fully connected layer, which takes as input the characteristics generated from a sentence by the LSTM layer and consequently detects the most appropriate label according to semantic and syntactic content. The probability that a sentence belongs to the smoking categories is calculated by the Softmax activation function.

#### Model architectures

Combining the different feature extraction methods with CNN-LSTM and the SE resulted in seven architectures: (1) Embedding with CNN-LSTM, (2) Embedding with SE, (3) Bert with CNN-LSTM, (4) Bert with SE, (5) Word2Vector with CNN-LSTM, (6) Word2Vector with SE, and (7) Count Vectorizer with SE. The details of these architectures are presented in Fig. 1, Sect. 4.

In this study, we chose not to employ the combination of Count Vectorizer with a CNN-LSTM architecture. The rationale behind the decision lies in the intrinsic design of the Count Vectorizer, which produces a bag-of-words representation, consequently discarding word order. CNN-LSTM architectures are specifically tailored to capture sequential patterns in data; therefore, using a bag-of-words representation compromises their primary advantage. Furthermore, the integration of CNN-LSTM introduces substantial complexity to the model. In the absence of sequential data to leverage the unique strengths of CNN-LSTM, alternative simpler models may potentially offer comparable or superior performance without the computational overhead of such intricate architectures.

### Model evaluation

To assess the detection performance of the created classifiers, several metrics were employed, including the receiver operating characteristics curve (ROC), area under the receiver operating characteristics curve (AU-ROC), Precision, Recall, F1-Score, and detection accuracy. These performance metrics are determined by searching for the values of true positive (TP), false positive (FP), false negative (FN), and true negative (TN). Detailed descriptions of the evaluation metrics used are presented in Table 3.

**Table 3:** Description of performance metrics and their formula. Area under receiver operating characteristics curve (AU-ROC)

Metric	Description	Formula
Precision	Precision, also known as Positive Predictive Value (PPV), is a performance measure that quantifies the proportion of predicted positive records that are actually true positives. Its primary purpose is to minimize the occurrence of false positives.	$Precision= \frac{TP}{TP+FP}$
Recall	Recall, also referred to as True Positive Rate (TPR), is a performance metric that assesses the sensitivity of a classifier. It measures the ability of the model to correctly identify positive samples. Recall is especially useful when the objective is to capture all positive samples and avoid false negatives.	$Recall \left(Sensitivity\right)= \frac{TP}{TP+FN}$
F1-Score	The F1-Score is measured by calculating the average of Precision and Recall. It provides insight into the classifier’s ability to identify positive records accurately. A higher F1-Score indicates superior performance of the classifier in the positive class. When dealing with imbalanced datasets of binary classifications, F1-Score can be a better metric to use than accuracy.	$F1-Score=2\times \frac{Precision \times Recall}{Precision+Recall}$
Detection Accuracy	The most commonly used metric to evaluate a classifier’s performance is detection accuracy, which measures how accurately the algorithm detects the actual class labels. Calculating detection accuracy is a quick way to assess the effectiveness of the detection model and overall performance. However, while it provides a general sense of the model’s performance, it may not provide detailed information on the classifier’s performance and may not be the best metric to consider in some cases.	$Accuracy= \frac{TP+TN}{TP+TN+FP+FN}$
AU-ROC	The AU-ROC is a performance metric that represents the area under the ROC curve, summarizing the overall performance of classifiers. It assumes that the errors of false positives and false negatives have equal importance. However, in medical situations, false negatives are usually considered more serious since those individuals are not detected by the test. False positives, on the other hand, can be retested to correct the classification. The ROC curve is a graphical representation of the trade-off between True Positive Rate (TPR) and False Positive Rate (FPR) for various threshold settings. It plots the cumulative distribution function of a defined probability distribution for both correctly and incorrectly classified events. TPR is shown on the y-axis, while FPR is on the x-axis.	$False Positive Rate= \frac{FP}{TN+FP}$

### Model explanation

Explainable Artificial Intelligence (XAI) techniques helps to explain the decisions made by machine learning models so that humans can understand. Ensuring that clinical staff and end users trust a machine learning model’s decisions requires making it’s reasoning process clear and comprehensible [37]. The local interpretable model-agnostic explanations (LIME) framework is one of the most extensively used XAI packages that enables classifiers to explain individual detection [38]. It explains a decision by locally approximating the classifier’s decision boundary in the given instance’s neighborhood. LIME builds locally linear models to explain the detection of a machine learning model. It corresponds to the rule-based regional explanations through the simplification category. Explanations through simplification build an entirely new model based on the trained machine learning model to be explained. The newly simplified model then attempts to optimize its similarity to its previous model functions while lowering complexity and maintaining comparable performance. As a result, after the machine learning decision is achieved, the LIME is used to assess the features’ importance and probabilities in the decision. As a result, we can determine the importance of the features in the decision input, which assists in interpreting the model outputs. We applied this technique to the models, which has the highest detection. Since the data are private and contain sensitive information, only the non-sensitive portions of the sentences are displayed in the examples.

## Results

### Dataset description

From the total cohort of patients examined on suspicion of lung cancer (*N* = 38,944), notes containing the two subheaders were available on 23,542 patients (59%). After removing duplicates and patients missing data on gender, the final cohort was reduced to 23,132 patients, each with multiple registrations (92,113 notes). Each note contained an average of 60 tokens, but the range of the token length varied between 1 and 1051. The annotation of the 23,132 patients with available notes resulted in the following distribution of smoking habits: 6121 (26%) Never-Smoker, 10,617 (46%) Former-Smoker and 6394 (28%) Active-Smoker. They were further pooled into binary labels of Non-Smoker (26%) and Smoker (74%), which is former and active smokers.

To validate the data annotation, the results were matched against the registrations in the Danish Lung Cancer Registry. From the 23,132 patients with EHR-annotated smoking status, 4719 had lung cancer. Among these, data on smoking status registered in the Danish Lung Cancer Registry was available on 4168 patients. In the registry 217 patients were listed as Non-Smoker, of which the EHR annotation was equivalent in 83% of the cases. The registration as Smoker was made on 3787 patients of which the EHR annotation was equivalent in 97% of cases. This was overall considered to be a high correlation between the results and acceptable validity of the manual annotation from free text.

### Binary classification

**Fig. 2:**
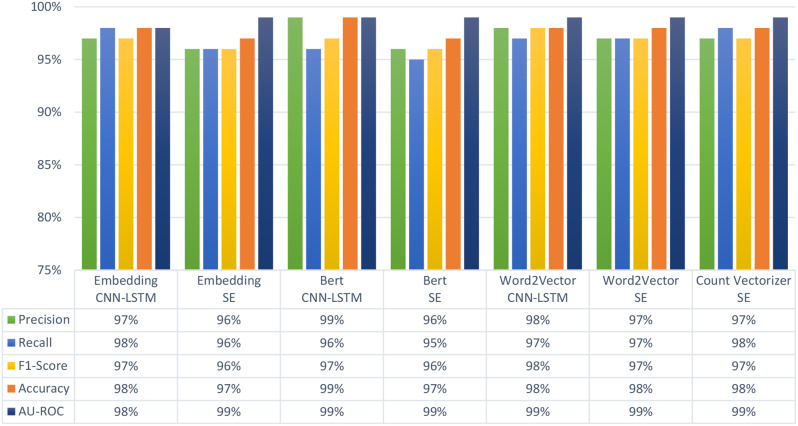
Average performance measures based on binary classification of the seven model architectures. CNN-LSTM: Convolutional neural network with a long short-term memory layer. SE: Stacking-Based Ensemble. BERT: Bidirectional Encoder Representations from Transformers. AU-ROC: Area under Receiver Operating Characteristic Curve

It is important to note that in terms of precision, recall, and F1-score, the SE-based architecture was low on average and class-specific performance. As presented in Fig. 2, BERT with SE and Embedding with SE achieved the worst results compared with the other feature extraction methods, in which the accuracy reached 97%. This might be due to high dimensionality, causing the SE to be less effective when compared to alternative methods. On the other hand, BERT with CNN-LSTM could achieve almost the highest overall accuracy and precision of 99% among all developed architecture. However, as shown in Table 4, BERT using CNN-LSTM shared the best precision of 99% with Embedding using the CNN-LSMT architecture for the Smoker class.

**Table 4:** Class-specific evaluation measures based on binary classification of the seven model architectures. CNN-LSTM: Convolutional neural network with a long short-term memory layer. SE: Stacking-Based Ensemble. BERT: Bidirectional Encoder Representations from Transformers

Performance binary							
	Embedding CNN-LSTM	Embedding SE	Bert CNN-LSTM	Bert SE	Word2Vector CNN-LSTM	Word2Vector SE	Count vectorizer SE
Non-Smoker							
Precision	94%	94%	99%	96%	97%	95%	95%
Recall	97%	95%	98%	92%	95%	96%	97%
F1-Score	96%	95%	97%	94%	96%	95%	96%
Smoker							
Precision	99%	98%	99%	97%	98%	98%	98%
Recall	98%	98%	100%	99%	99%	98%	98%
F1-Score	98%	98%	99%	98%	99%	98%	99%

In terms of recall, Embedding with CNN-LSTM and Count Vectorizer with SE achieved the highest precision of 98% as shown in Fig. 2. For the single class of Smoker, however, Bert with CNN-LSTM achieved the highest recall of 100% (Table 4). In terms of F1-Score, Word2Vector achieved the highest overall performance of 98%. As to F1-Score of a single class of Smoker, three architectures achieved the highest score of 99%, i.e., BERT with CNN-LSTM, Word2Vector with CNN-LSTM and Count Vectorizer with SE.

**Fig. 3:**
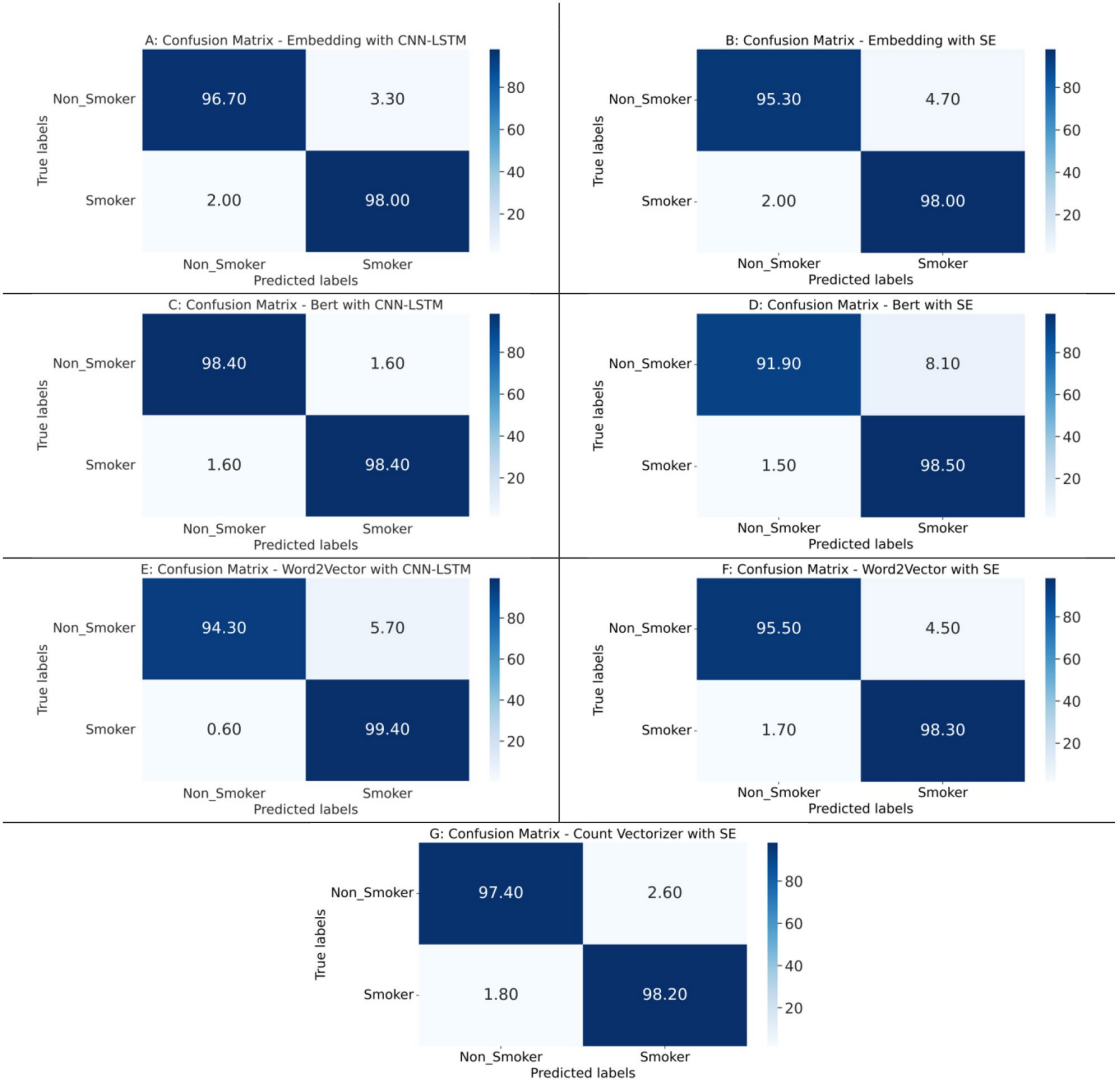
Confusion matrixes based on binary classification of all seven model architectures

Results based on confusion matrix (Fig. 3) indicates that Word2Vector with CNN-LSTM architecture had the best performance in terms of detecting Smoker class with a true detection rate of about 99%. BERT with CNN-LSTM architecture performed best in detecting Non-Smoker patients at a true detection rate of about 98%. The results of other machine learning classifiers including KNN, DT, RF, and XGBoost are presented in Supplementary Fig. 1 and Supplementary Table 1.

### Multiclass classification

**Fig. 4:**
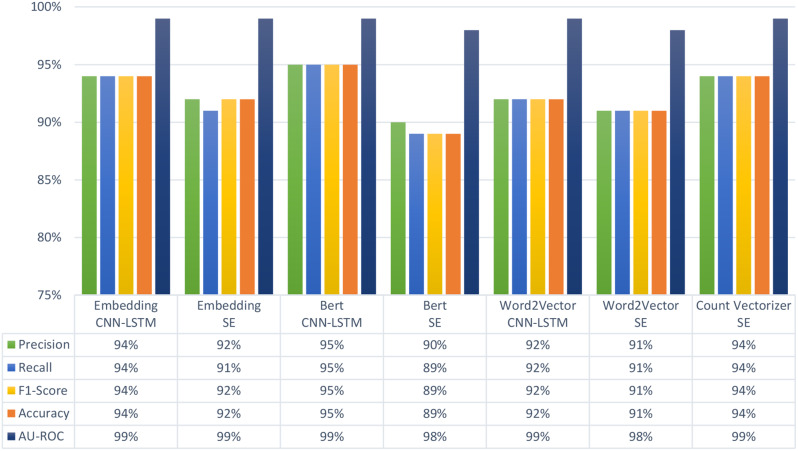
Average performance measures based on multiclass classification of the seven model architectures. CNN-LSTM: Convolutional neural network with a long short-term memory layer. SE: Stacking-Based Ensemble. BERT: Bidirectional Encoder Representations from Transformers. AU-ROC: Area under Receiver Operating Characteristic Curve

As presented in Fig. 4, BERT with SE had the lowest performance compared to the other feature extraction methods, in which the accuracy reached 89%. Contrarily, BERT with CNN-LSTM achieved the highest accuracy, precision, recall, F1-score, and AUC of 95%. This architecture also performed the best in most of the class specific outcomes. As presented in Table 5, BERT with CNN-LSTM had the highest performance for precision and F1-score of the Never-Smoker and Active-Smoker classes. In terms of precision, this architecture achieved 98% and 95% in the Never-Smoker and Active-Smoker classes, respectively. In terms of F1-score, it achieved 97% and 93% in the Never-Smoker and Active-Smoker classes, respectively.

**Table 5:** Class-specific evaluation measures based on multiclass classification of the seven model architectures. CNN-LSTM: Convolutional neural network with a long short-term memory layer. SE: Stacking-Based Ensemble. BERT: Bidirectional Encoder Representations from Transformers

Performance multiclass							
	Embedding CNN-LSTM	Embedding SE	Bert CNN-LSTM	Bert SE	Word2Vector CNN-LSTM	Word2Vector SE	Count vectorizer SE
Never-Smoker							
Precision	97%	95%	98%	96%	94%	95%	95%
Recall	97%	96%	97%	93%	96%	96%	98%
F1-Score	97%	96%	97%	94%	85%	95%	96%
Active-Smoker							
Precision	93%	89%	95%	85%	89%	87%	93%
Recall	91%	86%	91%	83%	87%	86%	91%
F1-Score	92%	88%	93%	84%	88%	86%	92%
Former-Smoker							
Precision	94%	91%	94%	88%	92%	90%	94%
Recall	95%	92%	97%	90%	92%	91%	94%
F1-Score	95%	92%	95%	94%	92%	91%	94%

Other architectures also achieved reasonable detection performances close to the performance of BERT with CNN-LSTM architecture. Embedding with CCN-LSTM and Count Vectorizer with SE achieved an overall accuracy of 94% (Fig. 4), which is only 1% lower than BERT with CNN-LSTM. Considering the results in Table 5, Embedding with CCN-LSTM and BERT with CNN-LSTM architecture achieved the highest precision and F1-scores of 94% and 95%, respectively, for the Former-Smoker class. In terms of recall, the results for each class varied. For the Never-Smoker class, Count Vectorizer with SE achieved the highest recall of 98%. For the Active-Smoker class, Embedding with CNN-LSTM, BERT with CNN-LSTM, and Count Vectorizer with SE achieved the highest recall of 91%. In the Former-Smoker class, BERT with CNN-LSTM achieved the highest recall of 97%.

**Fig. 5:**
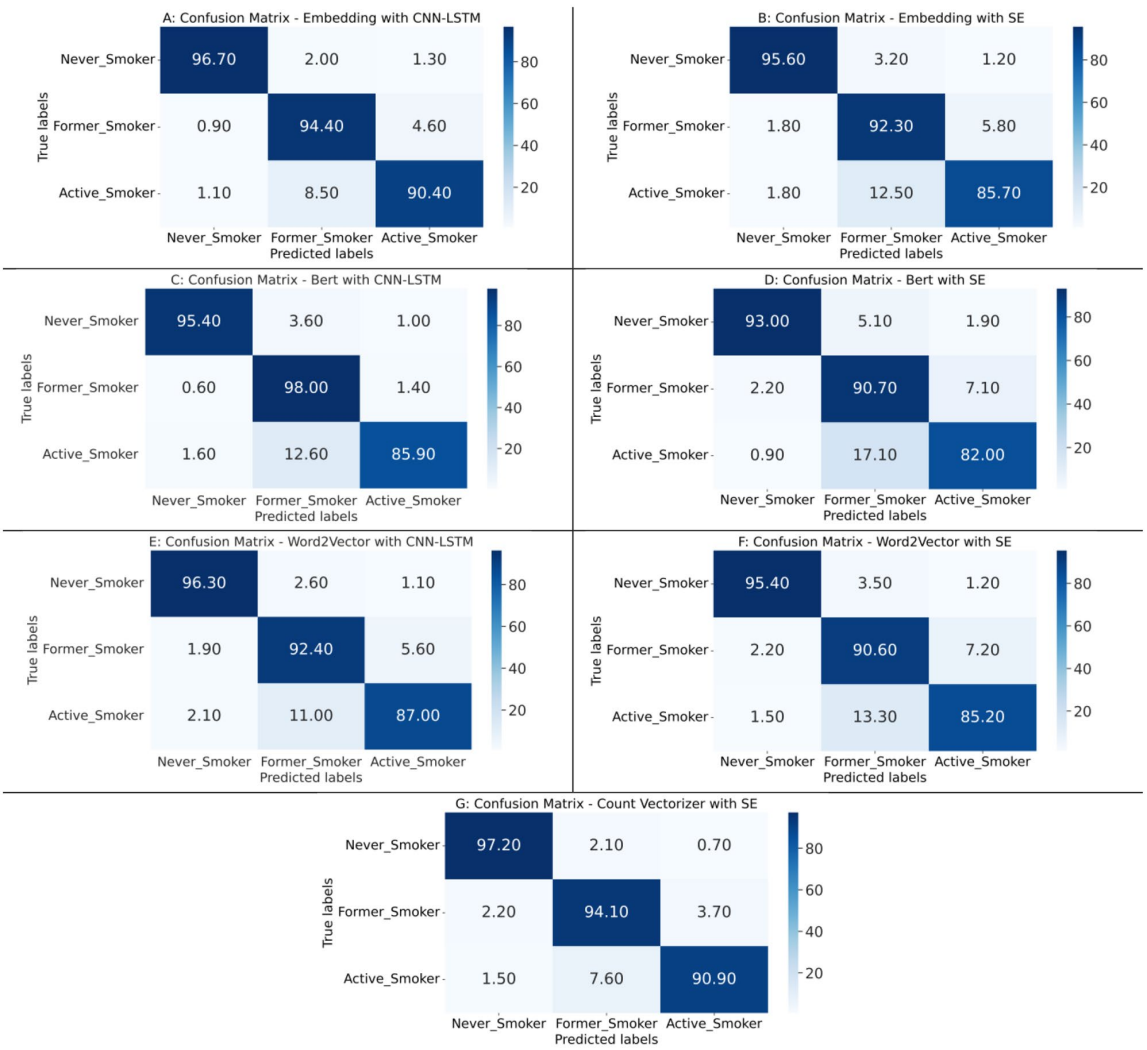
Confusion matrixes based on multiclass classification of the architectures of all seven models

Results derived from the confusion matrix reveal that the Embedding with CNN-LSTM and Count Vectorizer with SE architectures exhibited superior performance in detecting the Active-Smokers and Never-Smoker classes, yielding true detection rates of approximately 91% and 97%, respectively (Fig. 5). BERT with CNN-LSTM excelled in identifying samples from the Former-Smoker class, with a true detection rate of 98%. When accounting for the smallest discrepancy in detection rates across all classes, both the Embedding with CNN-LSTM and Count Vectorizer with SE architectures were the most consistent. This suggests a marginal difference of about 4% between the Former-Smoker and Active-Smoker classes, which is the narrowest gap observed across all architectures. The marginal difference between the Never-Smoker class and other classes in the Embedding with CNN-LSTM architecture presents the narrowest gap compared to all other architectures developed. The results of other machine learning classifier including KNN, DT, RF, and XGBoost are presented in Supplementary Fig. 2 and Supplementary Table 2.

### Post-hoc comparison of model architectures

**Fig. 6:**
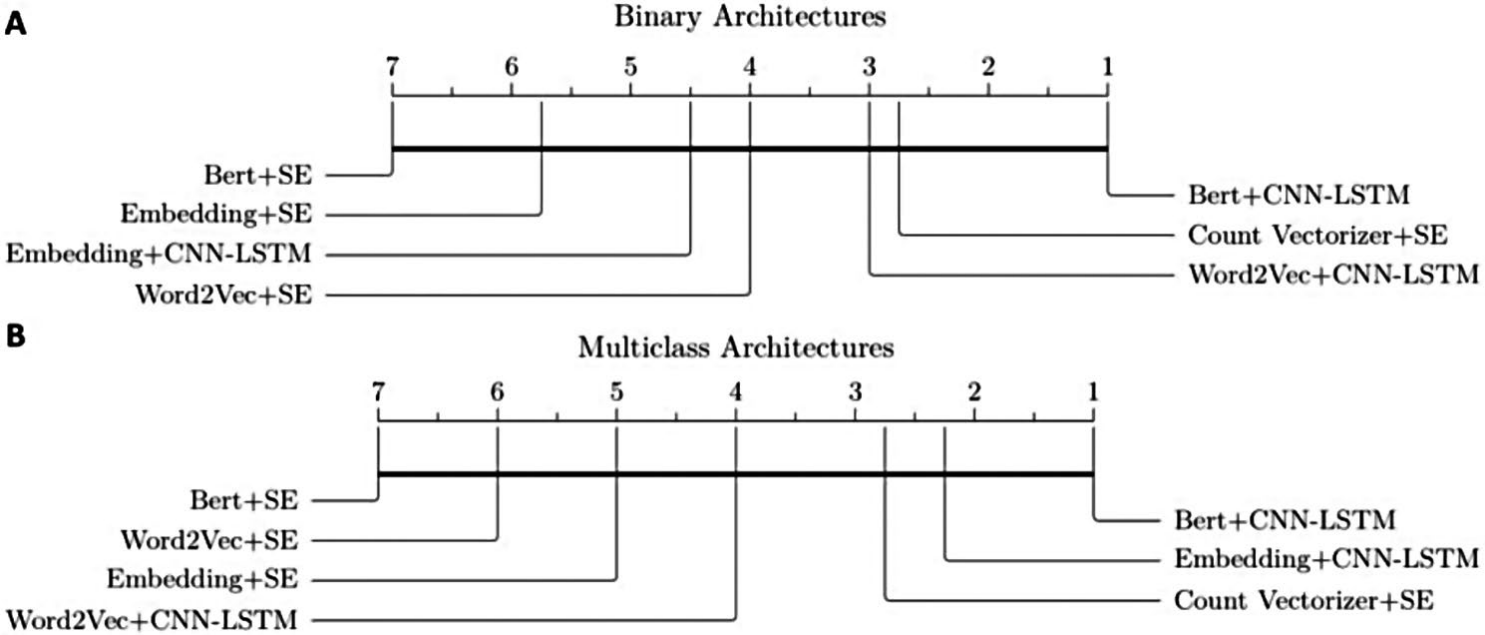
Results of the Friedman test and Nemenyi post-hoc test, α = 0.05

Since the results derived from detection performances and confusion matrices did not provide sufficient insight to determine the optimal model, we conducted a Friedman test on the mean of average results from the seven developed architectures. As shown in Fig. 6 there was no significant difference in average performance between the classifiers, neither concerning the binary (A) nor the multiclass architectures (B).

### XAI to explain detection model decisions

**Fig. 7:**
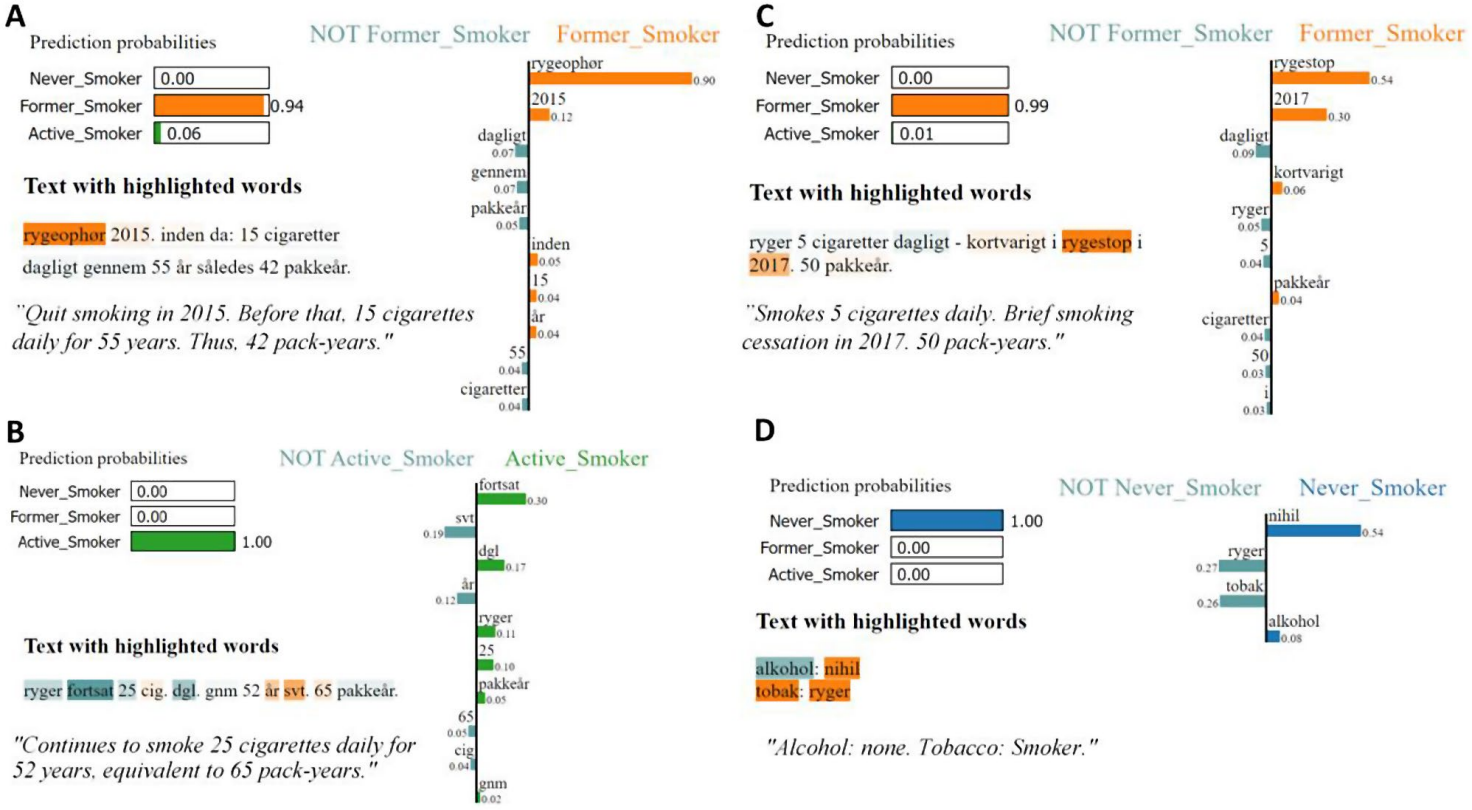
LIME plots representing the outcomes of multiclass classification of four distinct samples derived from Embedding with CNN-LSTM. **A**: Former-Smoker accurately detection with a 94% probability of being a Former-Smoker. **B**: Active-Smoker correctly detection with a 100% probability of being an Active-Smoker. **C**: Active-Smoker misclassified as a Former-Smoker. **D**: Smoker wrongly classified as a Non-Smoker.

The results indicate that classifying between ‘Former-Smoker’ and ‘Active-Smoker’ status is challenging, as the models occasionally underperformed in these categories. Nonetheless, the architecture of Embedding with CNN-LSTM reached a nearly optimal performance. In this section we explain the framework of the architecture utilizing the LIME technique as depicted in Fig. 6. All examples come with the original text and plots illustrating the importance of features for the detected class compared to the remaining two classes. Figure 7A displays the data on a Former-Smoker accurately detected with a probability of 94% of being categorized as a Former-Smoker. The key feature, “rygeophør” (smoking cessation), played a central role in assigning the case to the Former-Smoker category. Figure 7B presents the data of an Active-Smoker that was correctly detected with a probability of 100% as an Active-Smoker. This outcome was primarily influenced by the words “fortsat” (continued) and “dgl” (daily), which classified the patient into the Active-Smoker category. Figure 7C, however, portrays an Active-Smoker that was misclassified as a Former-Smoker, with a detected high probability of 99% of being a Former-Smoker and merely 1% of being an Active-Smoker. The words “rygestop” (smoking cessation) and “2017” contributed significantly to the detection, while the words “dagligt” (daily) and “ryger” (smoker) skewed the classification toward the Active-Smoker label. Figure 7D exhibits a Smoker incorrectly labeled as a Non-Smoker, due to the misinterpretation of the word “nihil” (nothing) within an alcohol assessment context.

## Discussion

Summary of findings.

We proposed effective detection NLP-based architectures for detection of smoking status using Danish EHRs. The data were collected from 23,132 patients who underwent examinations on suspicion of lung cancer. They were conducted at pulmonary departments in the Region of Southern Denmark from 2009 to 2018. Our proposed method encompassed the utilization of seven diverse model architectures developed through a combination of feature extraction techniques (embedding, BERT, Word2Vector, and count vectorizer), machine learning (SE) and deep learning (CNN-LSTM) models. We evaluated the performance of the architectures by examining various metrics for binary (Non-Smoker and Smoker) and multiclass (Never-Smoker, Active-Smoker, and Former-Smoker) classification tasks. Each metric focuses on a special aspect of the performance. Except for the AU-ROC, all metrics were constructed based on a confusion matrix (TP, FP, TN, and FN).

Given the complex nature of Danish language, particularly its compound word formation and unique syntactic structures, our proposed methodology was accurately designed to ensure the relevance and effectiveness of selected NLP pipeline in processing Danish language. The developed models and feature extractions were chosen for their robust linguistic capture capabilities, essential for the syntactic and morphological complexities of Danish. Adaptations included specialized preprocessing for Danish abbreviations and punctuation, and the fine-tuning of the BERT model with Danish EHR, enhancing its syntactic and semantic understanding of the language. The superior performance of the developed scenarios within our experimental validation highlights the success of these adaptations. Such outcomes not only validate our methodological choices but also underline the potential of our approach in advancing Danish language processing.

Performance metrics exhibited general similarity across the models, and post hoc tests revealed no significant differences when considering the mean of all outcomes. In terms of binary classification, however, the evaluations specific to each class indicated that BERT with CNN-LSTM outperformed the other models in all performance metrics.

In terms of multiclass classification, we observed that BERT with SE achieved the worst results compared with the other feature extraction methods in which the accuracy reached 89%. This was somehow expected due to the low amount of labeled data. BERT embeddings are high-dimensional vectors, which can lead to a large number of features when applied to the classical machine learning models. It resulted in high dimensionality causing the SE to become less efficient compared to other techniques.

On the other hand, the architecture of BERT with CNN-LSTM demonstrated overall superiority in terms of weighted average performance as well as class-specific performance metrics. It involves using BERT to generate contextual embeddings for the input text, passing them through a CNN layer to capture local features, and feeding the resulting features into an LSTM layer for sequential modeling and final classification. The superior performance of the BERT with CNN-LSTM architecture can be attributed to several key factors. Firstly, BERT, which is a state-of-the-art pre-trained language model, excels in capturing contextual information and semantic understanding from textual data. This enables it to extract intricate patterns and nuances in the EHRs related to smoking status, which can be highly context dependent. Furthermore, the combination of CNN and LSTM layers in this architecture allows for the effective extraction of both local and sequential features from the EHR text. CNNs are adept at capturing local patterns and features, while LSTMs excel at modeling sequential dependencies. The synergistic integration of these two components enables the model to capture a wide range of relevant information, from short-term textual features to long-term contextual dependencies, making it particularly well-suited for the nuanced task of smoking status identification. The combined approach helps the model effectively capture both global contextual information and local sequential patterns, resulting in improved performance in text classification tasks compared to using BERT with classic machine learning algorithms.

However, we believe that the Embedding with CNN-LSTM demonstrated the optimal results since the discrepancy in detection rates across all classes based on confusion matrix was the narrowest gap observed across the developed architectures. The Embedding with CNN-LSTM architecture exhibited more consistent detection rates across all classes compared to BERT with CNN-LSTM. This approach, with its straightforward embeddings, ensures efficient capture of semantic meanings, leading to faster training and reduced computational demands. Moreover, when tailored to specific datasets, the embeddings can potentially offer more aligned representations for the task at hand.

The consistent detection rates exhibited by the Embedding with CNN-LSTM architecture compared to BERT with CNN-LSTM can be attributed to its more structured feature representation, simpler model complexity, and potential alignment with the dataset’s characteristics. The use of word embeddings facilitates a focused representation of text data, aiding in the consistent identification of smoking-related terms across various classes. Additionally, the Embedding with CNN-LSTM’s relative simplicity may contribute to improved generalization across classes, particularly in the presence of class imbalances. This suggests that the architecture’s suitability for the dataset, combined with effective hyperparameter tuning, plays a crucial role in achieving stable and reliable detection rates across all classes. Hence, for the collected dataset in this study and the classification goals to detect smokers (Never-Smoker, Former-Smoker, Active-Smoker), the Embedding with CNN-LSTM architecture might be the more adaptable and optimal choice.

To provide additional insight into the interpretability of our results, we explored LIME-plots from the Embedding with CNN-LSTM architecture. Notably, these plots unveiled clinically relevant top features associated with each specific class. The utilization of explainable AI methods, notably the LIME, in the developed NLP pipeline, plays a pivotal role in enhancing the interpretability and trustworthiness of our smoking status identification process within the complex landscape of EHRs. With the natural complexity of EHR data, it is essential that our AI model’s decision-making is transparent and understandable to healthcare professionals. LIME enables us to provide detailed, human-readable explanations for each prediction, highlighting the most influential features and factors that led to a specific outcome. This not only empowers clinicians to gain deeper insights into the model’s reasoning but also allows them to validate the models’ decisions against their domain expertise. By bridging the gap between AI-driven predictions and clinical understanding, the explainable AI methods contribute significantly to the credibility and reliability of our smoking status identification system in the EHR environment, ultimately adding greater confidence in its utility and accuracy. The results were discussed with domain experts, who were in favor of a balanced performance across all classes in the dataset.

### Comparison to previous study results

Different studies have evaluated the application of NLP based on machine learning and deep learning techniques for the detection of smoking status through EHRs with different languages [12, 14, 39, 40]. Rajendran et al. developed a binary and multiclass classification model using English EHRs from the United States [14]. The model incorporated a CNN that utilized both a word-embedding layer pre-trained from the Google news corpus and a word2vec model, resulting in superior performance compared to conventional machine learning methods. The binary classification achieved an F1-measure of 85%, while the multiclass classification reached 68% for smoking status identification. Bae et al. developed a multiclass classification model using Korean and English EHR data extracted from 4711 clinical notes [39]. The most effective model employed an unsupervised keyword extraction technique in combination with a linear support vector machine, achieving an impressive F1-score of 91% for multiclass classification. Of note, both studies encountered challenges due to limited data availability and the extensive length of patient notes. Additionally, the Korean study faced limitations in terms of the relevant corpus available for pre-training, which necessitated the use of seed keywords pre-defined by clinicians for the keyword extraction method.

To the best of our knowledge, the most comparable study to ours is one based on Swedish EHR notes [40]. It developed classic machine learning models to classify smoking status into Current-Smoker, Ex-Smoker, Non-Smoker, and Unknown. Among the 32 developed detection models, support vector machine achieved the highest F1-score of 98%. The authors did not present the performance of developed models for each of the classes, which makes it difficult to understand the ability of models in different classes. Also, they did not consider any feature extraction method to transform the text into features and capture the essential information from the text. Consequently, the reasons for models’ decisions were not presented.

**Limitation and Future Work**

To the best of our knowledge this study represents the first exploration of a Danish NLP-model derived from a sizable dataset of manually annotated EHR-notes, but it has some limitations. It is important to acknowledge that the models are based on constrained input data. We exclusively considered text from the relatively short and simplistic subfield associated with smoking and risk factors in the EHR systems. Applying the established models on the complete EHR note is unquestionably bound to result in a performance decrease. Nevertheless, it is worth noting that the current Danish hospital systems store information on smoking status and other risk factors in a sub-header format similar to the structure observed in this dataset.

Another limitation pertains to the absence of an “unknown” category. Following the initial data annotation process, patients with unknown smoking status were further evaluated using additional notes. Ultimately, we selected the note containing the most detailed information on smoking status, resulting in the complete exclusion of the unknown category. This, however, represents a potential drawback since the model was not trained to classify “unknown” smoking status. Finally, it would be ideal to expand the model to include more detailed information on smoking status such as smoking duration and intensity. Incorporating these factors into a model would be relevant when determining eligibility for lung cancer screening. This would require a higher standard of quality and standardization in documenting smoking status compared to the current practices.

Based on the findings of this study, we plan to further explore the potential of this algorithm on longer EHR-notes without limitations to the subfield relevant to smoking. It would be valuable to incorporate free text from general practice to identify patients at risk of lung cancer or other chronic diseases where smoking status is a significant risk factor. However, data annotation remains a time-consuming task, and the size of the dataset may be limited by this factor when dealing with larger patient notes. Additionally, there is potential to annotate other risk factors, such as alcohol consumption, to expand the current model to different outcomes beyond smoking.

### Clinical perspectives

To the best of our knowledge, this is the first model based on Danish EHR data. Despite its limitations, the current model holds potential for application to Danish EHR data acquired at a hospital level. The ability to extract smoking status directly from free-text material would be highly advantageous, given that smoking status is a crucial risk factor for various acute and chronic illnesses. Having such information readily available for large patient populations allows for further investigation, as this variable is typically only accessible for specific populations such as patients with lung cancer or coronary heart disease. The incorporation of explainable AI, specifically LIME plots, opens possibilities for enhancing future models by identifying potential systematic errors. Additionally, it offers valuable insights into predictions, a crucial aspect for responsible clinicians. In addition to its potential in advancing research, this model could also find utility in screening scenarios, providing valuable information for risk assessment tools.

## Conclusion

We present the outcomes of a novel model capable of categorizing the smoking status of patients using Danish EHRs. By combining a transformer with a convolutional neural network, specifically BERT with CNN-LSTM, we achieved a remarkable performance, with low discrepancy in detection rates across all classes. This outcome accentuates the promising possibility of classifying smoking status based on unstructured free text data. The availability of comprehensive and precise information on smoking habits could potentially prove advantageous in future research endeavors. Moreover, it can aid in identifying high-risk individuals who are eligible for screening programs such as those aimed at detecting lung cancer.

### Electronic supplementary material

Below is the link to the electronic supplementary material.

Supplementary Material 1

## Data Availability

The dataset used for this study is not publicly available due to the possibility of compromising individual privacy but is available from the corresponding author on reasonable request.

## Abbreviations

NLP: Natural Language Processing
EHRs: Electronic Health Records
CNN-LSTM: Convolutional Neural Network with a long short-term memory layer
KNN: K-Nearest Neighbors
DT: Decision Tree
XG-Boost: Extreme Gradient Boosting
RF: Random Forest
SE: Stacking-based Ensemble
AU-ROC: Area Under the Receiver Operating Characteristics Curve
LIME: Local interpretable model-agnostic explanations
BERT: Bidirectional Encoder Representations from Transformers
TP: True Positive
TN: True Negative
FP: False Positive
FN: False Negative

## References

[1] de Boer AR et al. Data mining to retrieve smoking status from electronic health records in general practice. Eur Hear Journal-Digital Heal, 2022. 10.1093/ehjdh/ztac031 PMC9707867 36712169

[2] Roth G, Global Burden of Disease Collaborative Network. Global Burden of Disease Study 2017 (GBD 2017) Results. Seattle, United States: Institute for Health Metrics and Evaluation (IHME), 2018, *Lancet*, vol. 392, pp. 1736–1788, 2018.

[3] Malhotra J, Malvezzi M, Negri E, Vecchia CL, Boffetta P (2016). Risk factors for lung cancer worldwide. Eur Respir J.

[4] Lange P et al. Danish Register of chronic obstructive pulmonary disease. Clin Epidemiol, pp. 673–8, 2016. 10.2147/CLEP.S99489 PMC5094652 27822114

[5] Schmidt M (2019). The Danish health care system and epidemiological research: from health care contacts to database records. Clin Epidemiol.

[6] Afzal Z. Text mining to support knowledge discovery from electronic health records, *Erasmus University Rotterdam*, 2018. http://hdl.handle.net/1765/105993 (accessed Oct. 03, 2023).

[7] Groenhof TKJ (2020). Data mining information from electronic health records produced high yield and accuracy for current smoking status. J Clin Epidemiol.

[8] Liddy ED. Natural language processing, in *Encyclopedia of Library and Information Science, 2nd Ed. NY. Marcel Decker, Inc*, 2001.

[9] Byrd RJ, Steinhubl SR, Sun J, Ebadollahi S, Stewart WF (2014). Automatic identification of heart failure diagnostic criteria, using text analysis of clinical notes from electronic health records. Int J Med Inf.

[10] Sarker A, Gonzalez G (2015). Portable automatic text classification for adverse drug reaction detection via multi-corpus training. J Biomed Inf.

[11] Koleck TA, Dreisbach C, Bourne PE, Bakken S (2019). Natural language processing of symptoms documented in free-text narratives of electronic health records: a systematic review. J Am Med.

[12] Uzuner Ö, Goldstein I, Luo Y, Kohane I (2008). Identifying patient smoking status from medical discharge records. J Am Med Inf Assoc.

[13] Li I (2022). Neural natural language processing for unstructured data in electronic health records: a review. Comput Sci Rev.

[14] Rajendran S, Topaloglu U. Extracting smoking status from electronic health records using NLP and deep learning, *AMIA Summits Transl. Sci. Proc*, vol. 2020, p. 507, 2020. PMC7233082 32477672

[15] Devlin J, Chang M-W, Lee K, Toutanova K. Bert: Pre-training of deep bidirectional transformers for language understanding, *arXiv Prepr. arXiv1810.04805*, 2018.

[16] Devlin M-W, Jacob and, Chang O, Sourcing BERT. State-of-the-Art Pre-training for Natural Language Processing, 2018. https://blog.research.google/2018/11/open-sourcing-bert-state-of-art-pre.html (accessed Oct. 03, 2023).

[17] Wonsild F, Møller MG. Danish Clinical Event Extraction Developing a clinical event extraction system for electronic health records using deep learning and active learning, 2020, [Online]. Available: https://api.semanticscholar.org/CorpusID:220267240.

[18] Hvingelby R, Pauli AB, Barrett M, Rosted C, Lidegaard LM, Søgaard A. DaNE: A named entity resource for danish, in *Proceedings of the 12th Language Resources and Evaluation Conference*, 2020, pp. 4597–4604.

[19] Certainly. Certainly has trained the most advanced Danish BERT model to date, 2020. https://certainly.io/blog/danish-bert-model/ (accessed Oct. 03, 2023).

[20] Derczynski L et al. The Danish Gigaword Corpus, in *Proceedings of the 23rd Nordic Conference on Computational Linguistics (NoDaLiDa)*, 2021, pp. 413–421.

[21] Kirkedal A, Plank B, Derczynski L, Schluter N. The Lacunae of Danish Natural Language Processing, in *Proceedings of the 22nd Nordic Conference on Computational Linguistics*, 2019, pp. 356–362. [Online]. Available: https://aclanthology.org/W19-6141.

[22] Tjoa E, Guan C (2020). A survey on explainable artificial intelligence (xai): toward medical xai. IEEE Trans Neural Networks Learn Syst.

[23] Zini JE, Awad M (2022). On the explainability of natural language processing deep models. ACM Comput Surv.

[24] Henriksen MB (2023). A collection of multiregistry data on patients at high risk of lung cancer—a Danish retrospective cohort study of nearly 40,000 patients. Transl Lung Cancer Res.

[25] U.S. Department of Health and Human Services. National Cancer Institute Dictionary of Cancer Terms, 2024. https://www.cancer.gov/publications/dictionaries/cancer-terms/def/pack-year (accessed Apr. 04, 2024).

[26] Jurafsky D, Martin JH. Speech and Language Processing: An Introduction to Natural Language Processing, Computational Linguistics, and Speech Recognition.

[27] Selva Birunda S, Kanniga Devi R. A review on word embedding techniques for text classification, *Innov. Data Commun. Technol. Appl. Proc. ICIDCA 2020*, pp. 267–281, 2021.

[28] Manning C, Schutze H. *Foundations of statistical natural language processing*. MIT press, 1999. [Online]. Available: https://doc.lagout.org/science/0_ComputerScience/2_Algorithms/Statistical Natural Language Processing.pdf.

[29] Mikolov T, Chen K, Corrado G, Dean J. Efficient estimation of word representations in vector space, *arXiv Prepr. arXiv1301.3781*, 2013.

[30] Wolpert DH (1992). Stacked generalization. Neural Netw.

[31] Mucherino A, Papajorgji PJ, Pardalos PM, Mucherino A, Papajorgji PJ, Pardalos PM. K-nearest neighbor classification. Data Min Agric, pp. 83–106, 2009.

[32] Quinlan JR (1986). Induction of decision trees. Mach Learn.

[33] Rigatti SJ (2017). Random forest. J Insur Med.

[34] Bentéjac C, Csörg\Ho A, Mart\’\inez-Muñoz G (2021). A comparative analysis of gradient boosting algorithms. Artif Intell Rev.

[35] Tolles J, Meurer WJ (2016). Logistic regression: relating patient characteristics to outcomes. JAMA.

[36] Fang Z, Wang Y, Peng L, Hong H (2021). A comparative study of heterogeneous ensemble-learning techniques for landslide susceptibility mapping. Int J Geogr Inf Sci.

[37] Jain R, Ciravegna G, Barbiero P, Giannini F, Buffelli D, Lio P. Extending Logic Explained Networks to Text Classification, *arXiv Prepr. arXiv2211.09732*, 2022.

[38] Ribeiro MT, Singh S, Guestrin C. ‘ Why should i trust you?’ Explaining the predictions of any classifier, in *Proceedings of the 22nd ACM SIGKDD international conference on knowledge discovery and data mining*, 2016, pp. 1135–1144.

[39] Bae YS (2021). Keyword extraction algorithm for classifying smoking status from Unstructured Bilingual Electronic Health Records Based on Natural Language Processing. Appl Sci.

[40] Caccamisi A, Jørgensen L, Dalianis H, Rosenlund M. Natural language processing and machine learning to enable automatic extraction and classification of patients’ smoking status from electronic medical records., *Ups. J. Med. Sci*, vol. 125, no. 4, pp. 316–324, Nov. 2020, 10.1080/03009734.2020.1792010. 10.1080/03009734.2020.1792010 PMC7594865 32696698

