# Supplementary material for: Identification of patients’ smoking status using an explainable AI approach: a Danish electronic health records case study
Source: BMC Med Res Methodol. 2024 May 17;24:114. doi: 10.1186/s12874-024-02231-4 (PMC11100078; doi:10.1186/s12874-024-02231-4)
Supplement: Supplementary file 1 — Supplementary Material 1 [file 12874_2024_2231_MOESM1_ESM.docx]

# Binary Classification

This section presents the results obtained from various machine learning classifiers: KNN, DT, RF, and XGBoost. These classifiers were developed utilizing features extracted through different feature extraction methods to classify samples into 'Smoker' and 'Non-Smoker' classes.

| Figure 1. Confusion matrixes based on binary classification of all developed models. | |
| --- | --- |
| Machine learning models with Word2Vector | |
| 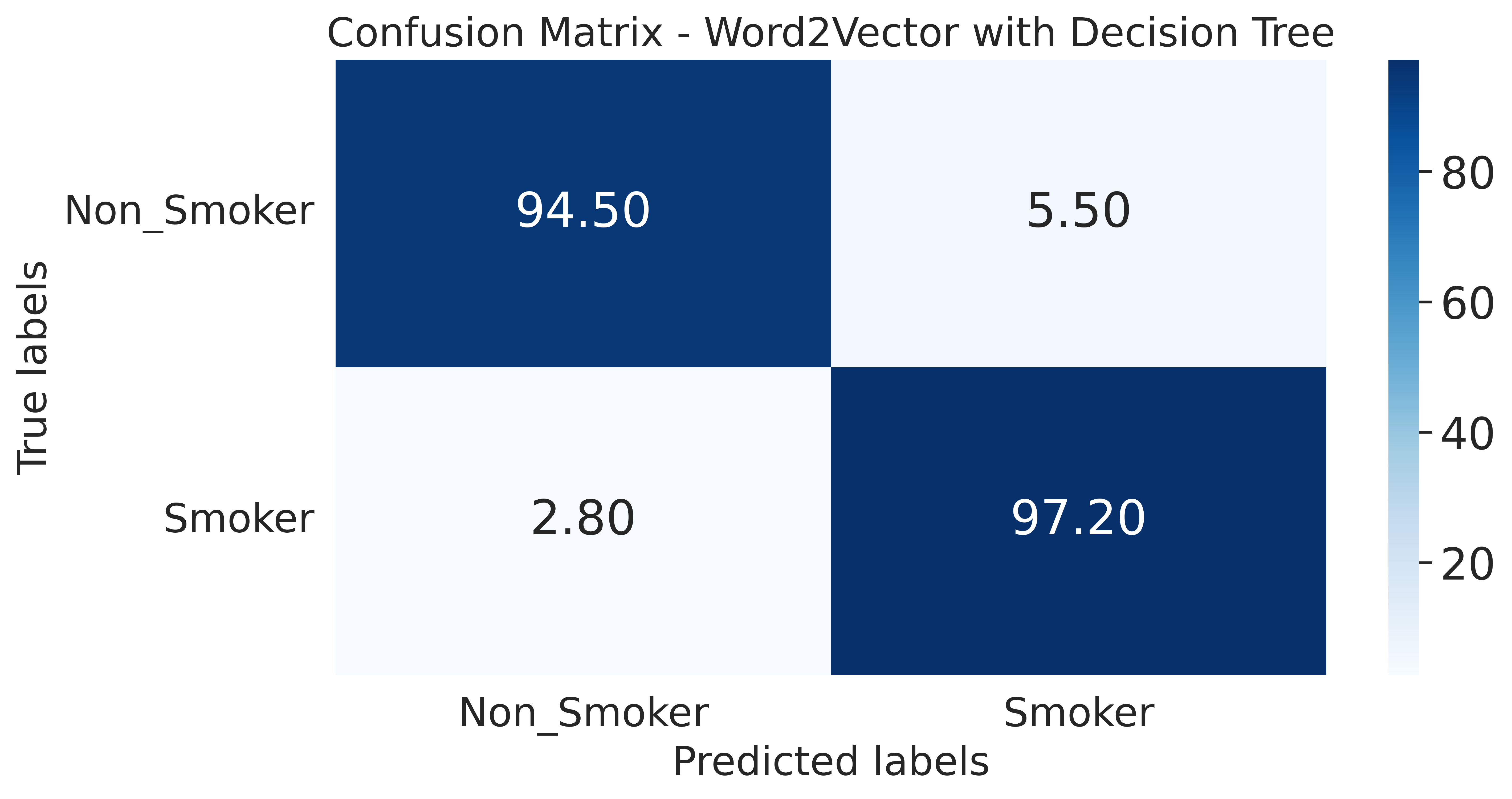 | 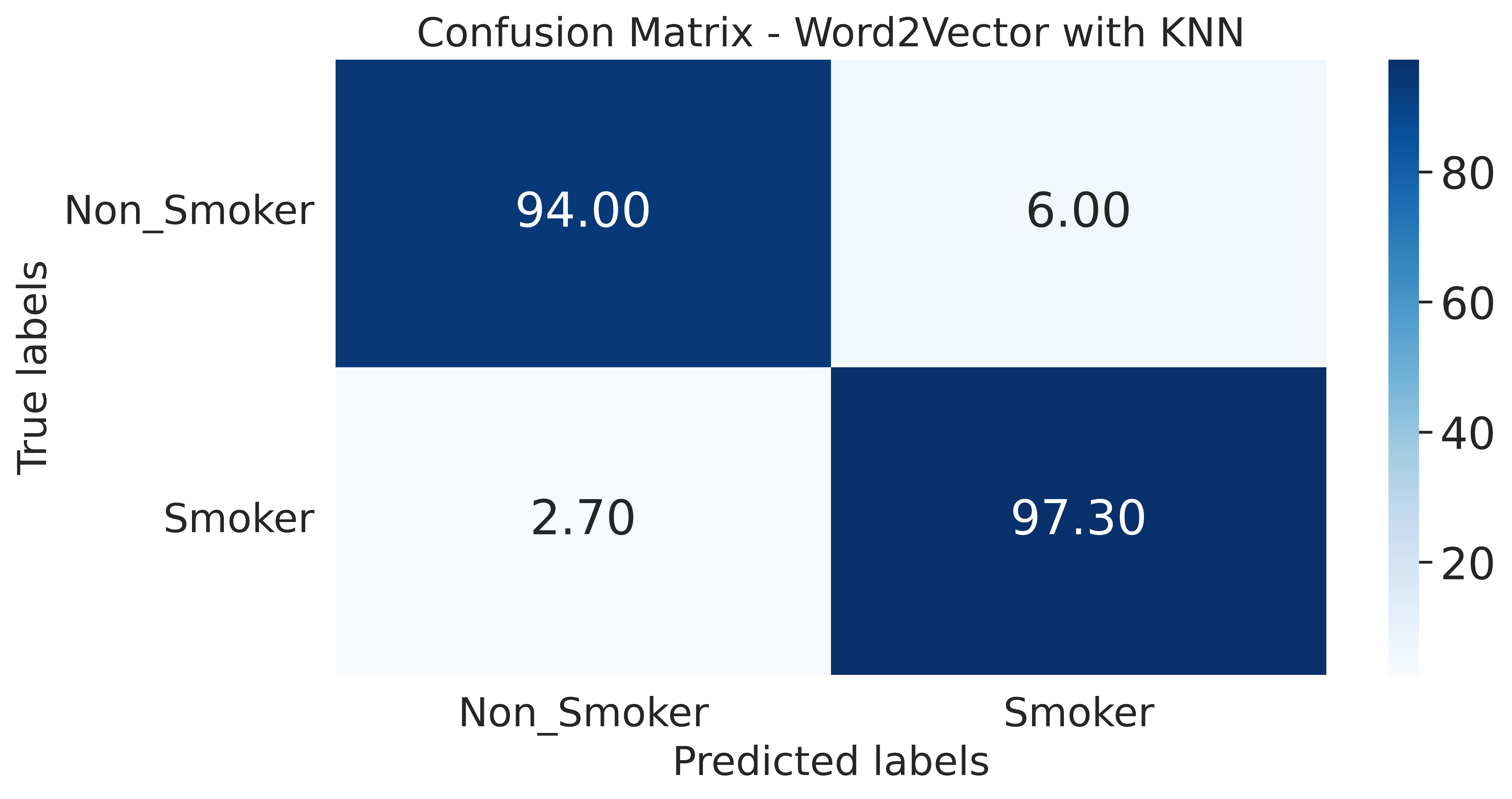 |
| 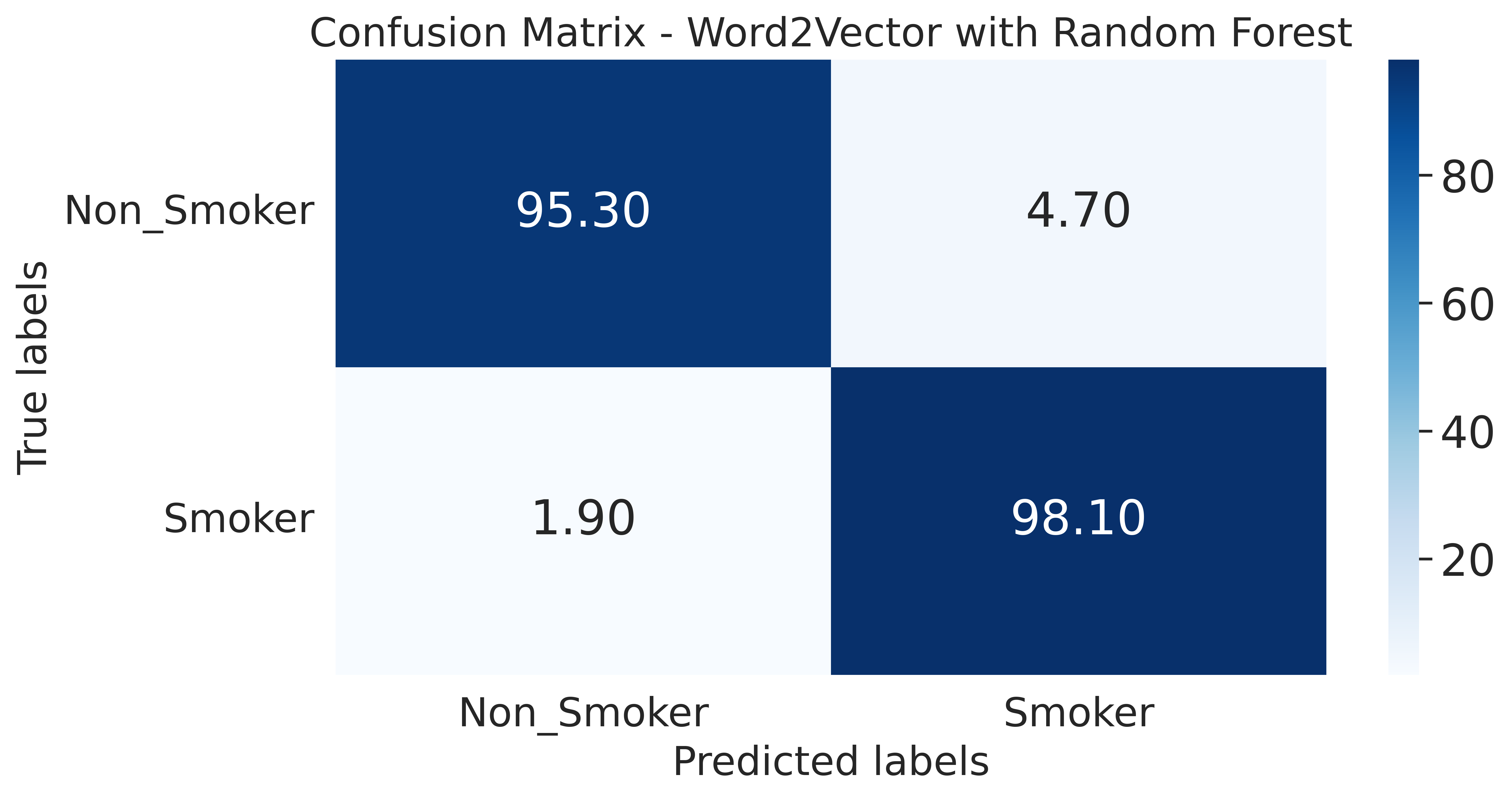 | 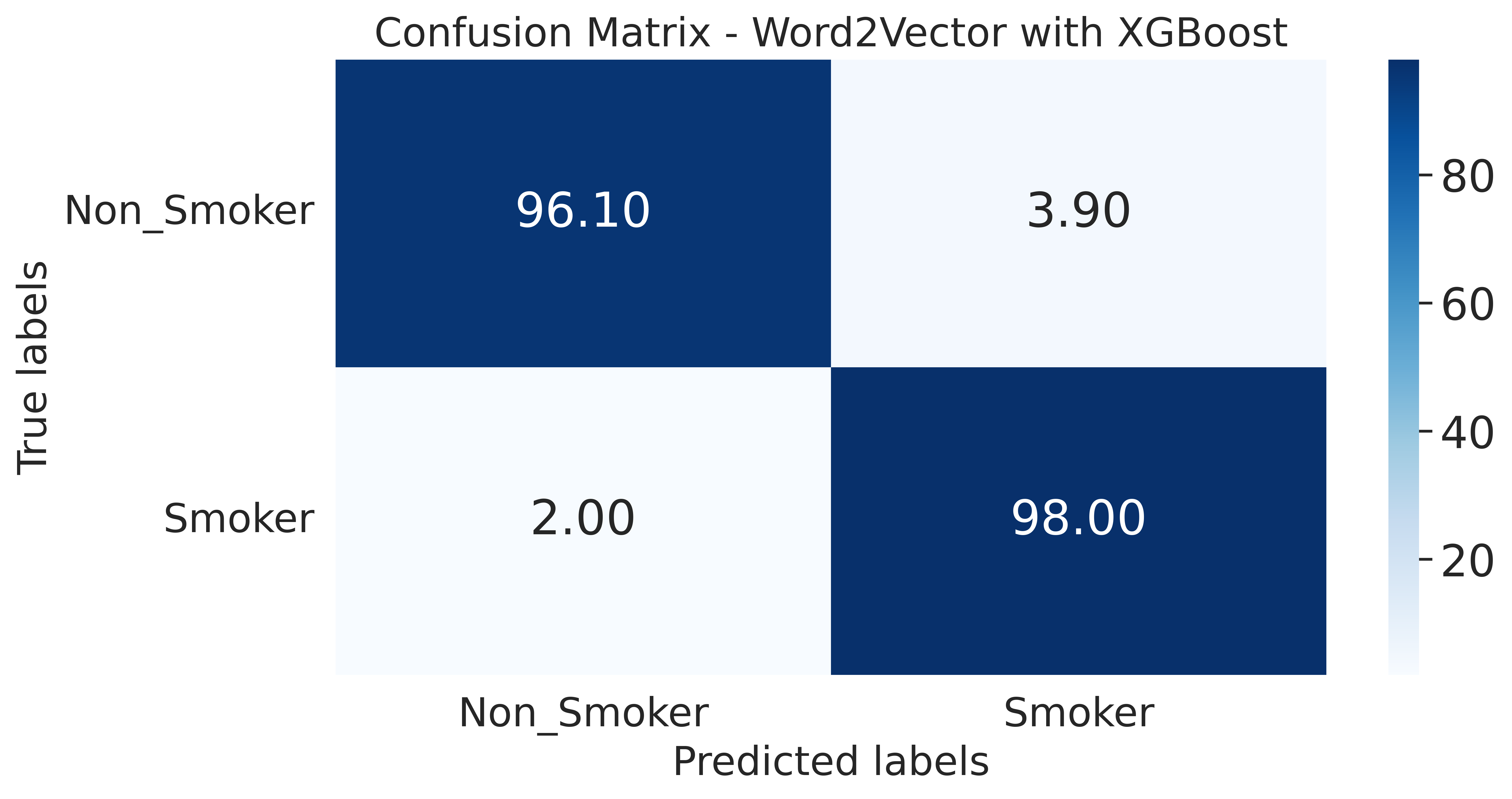 |
|  |  |
| Machine learning models with Embedding | |
| 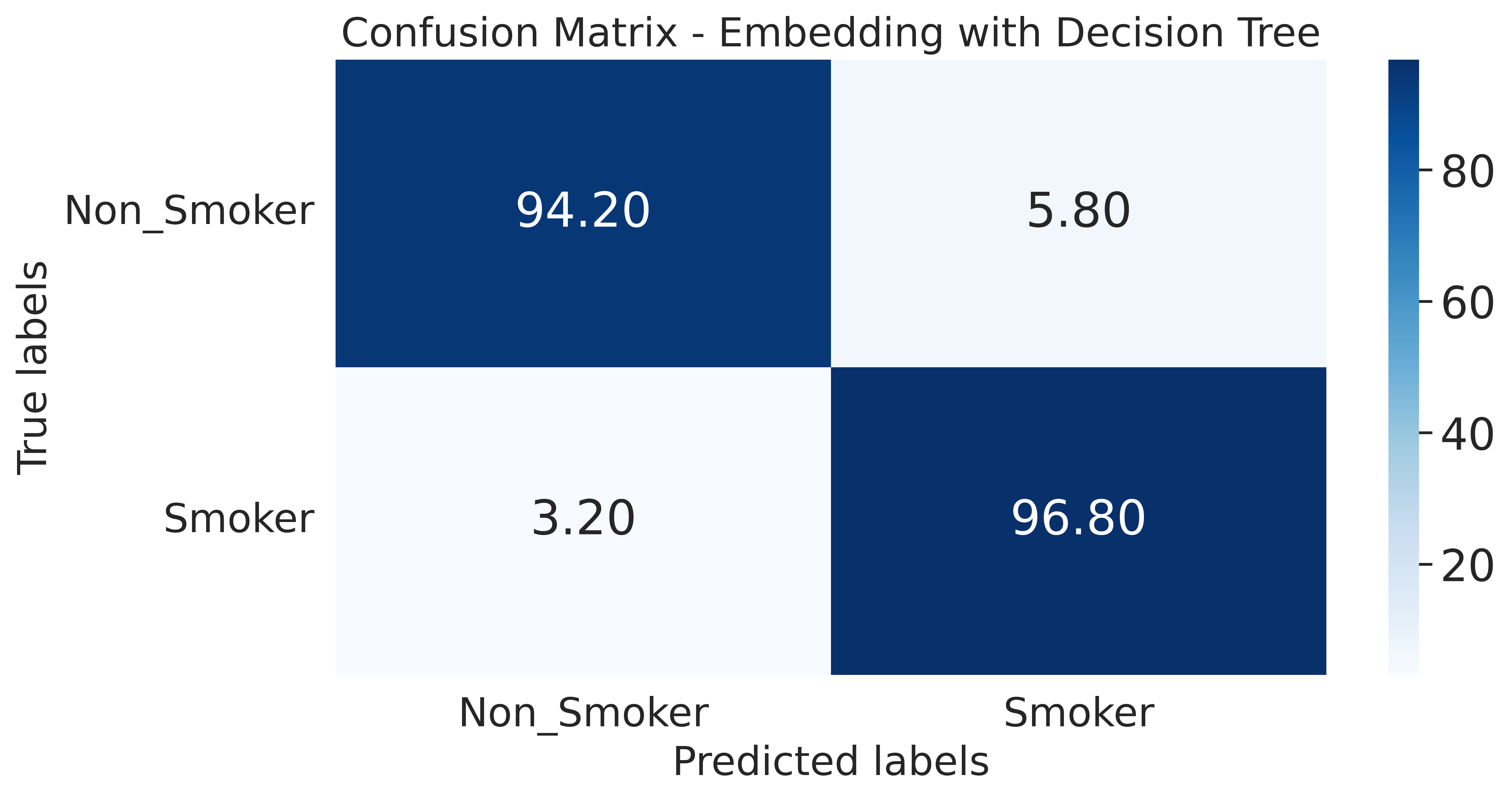 | 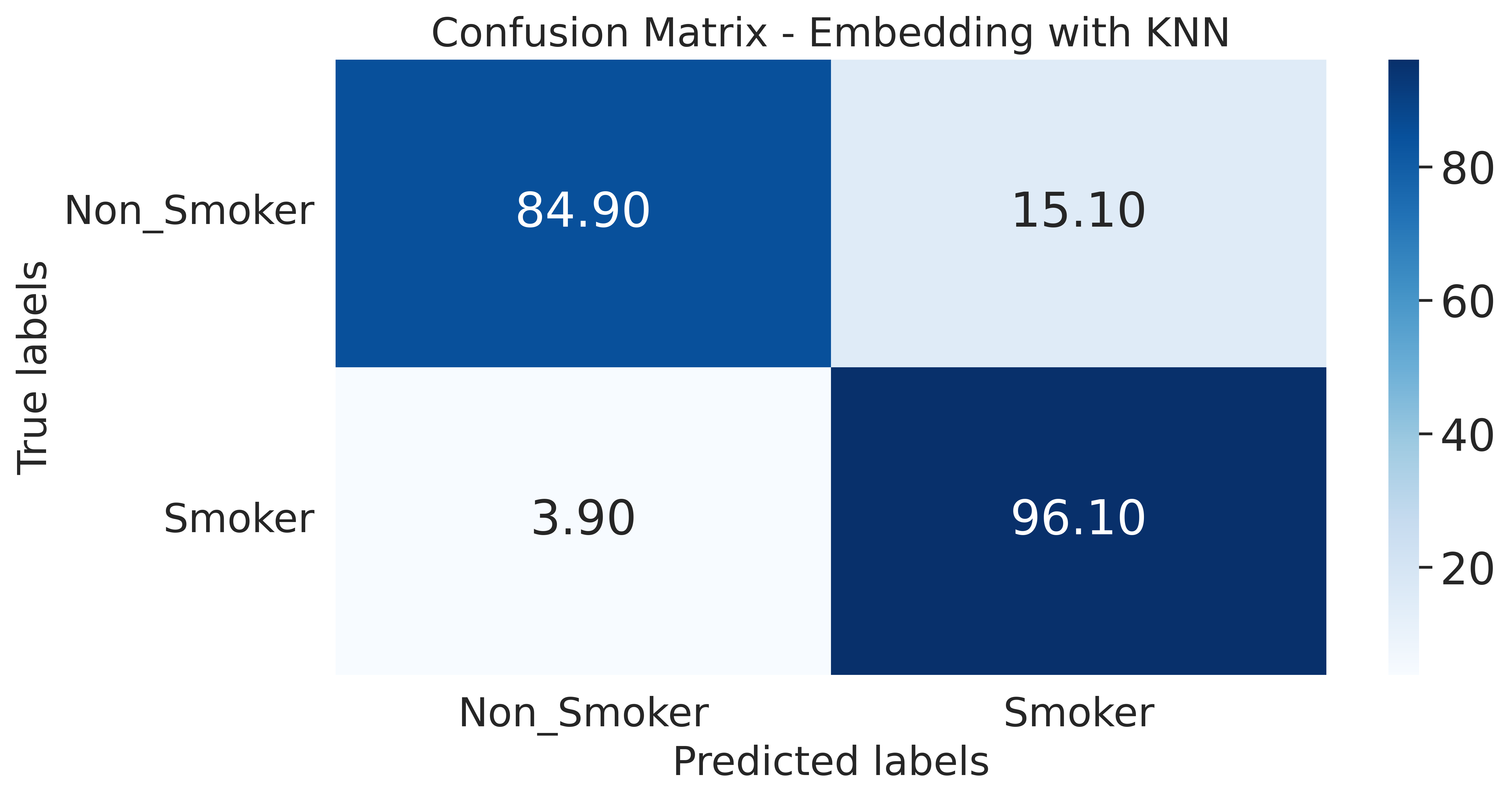 |
| 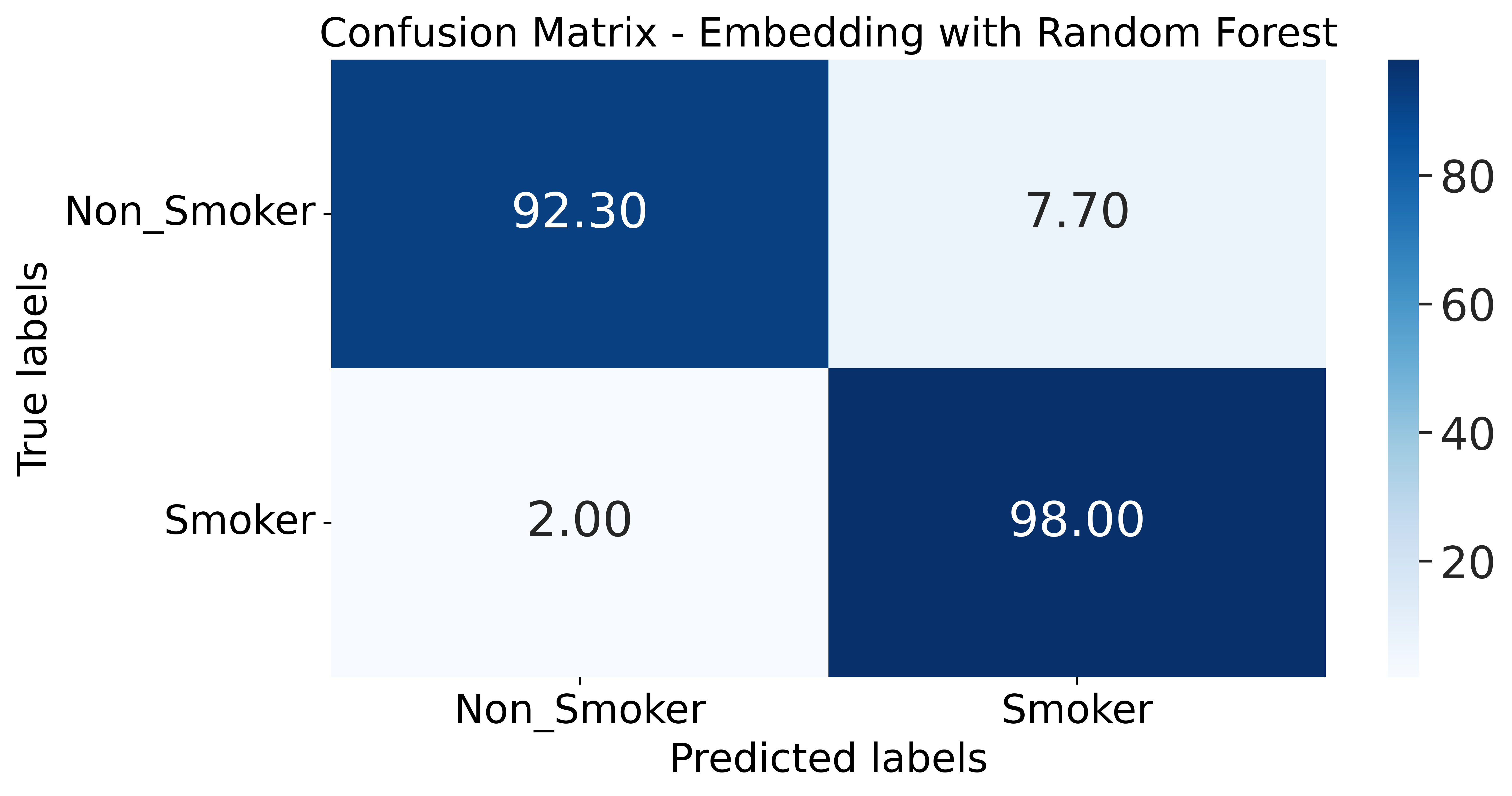 | 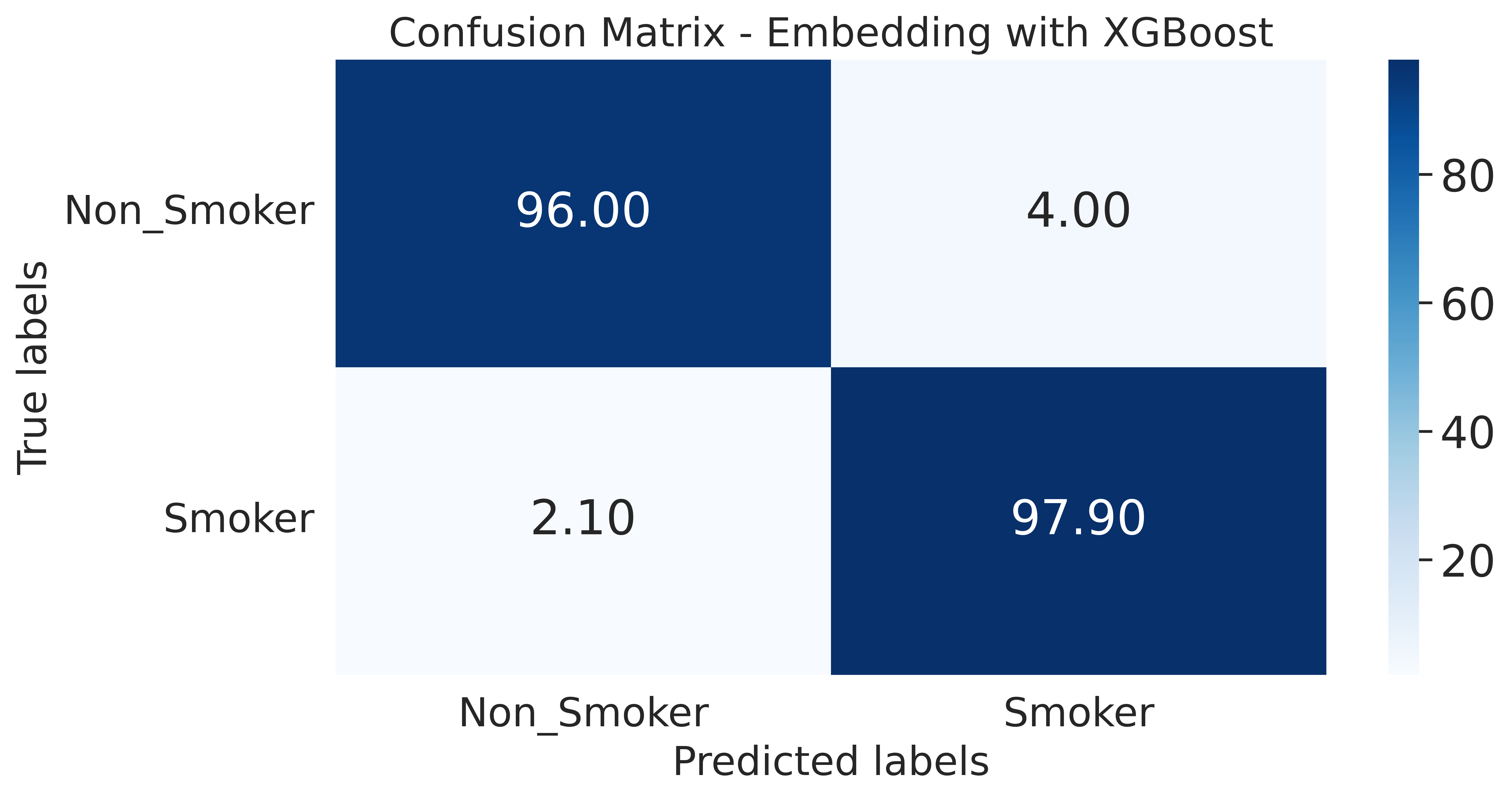 |
|  |  |
| Machine learning models with Bert | |
| 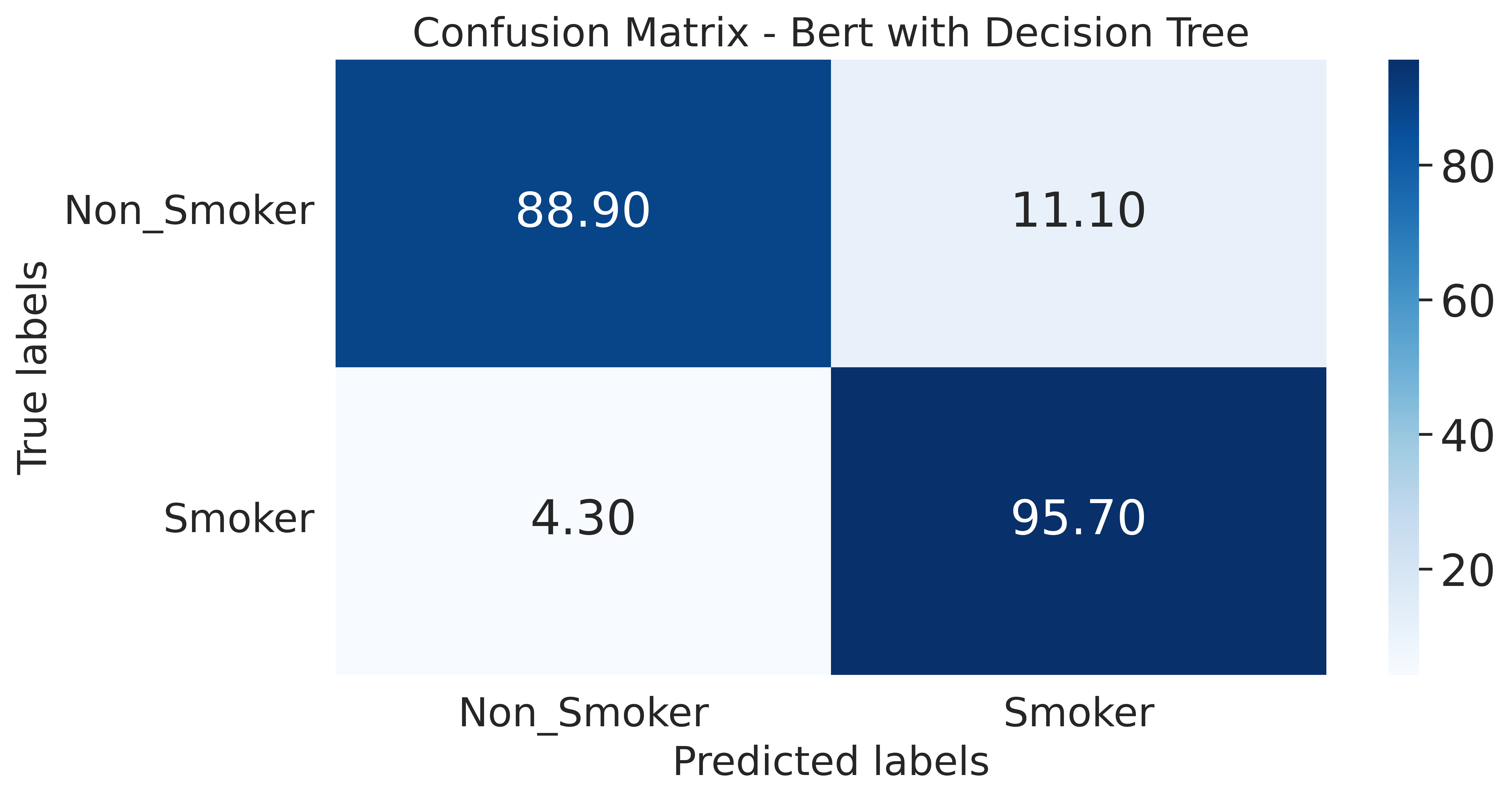 | 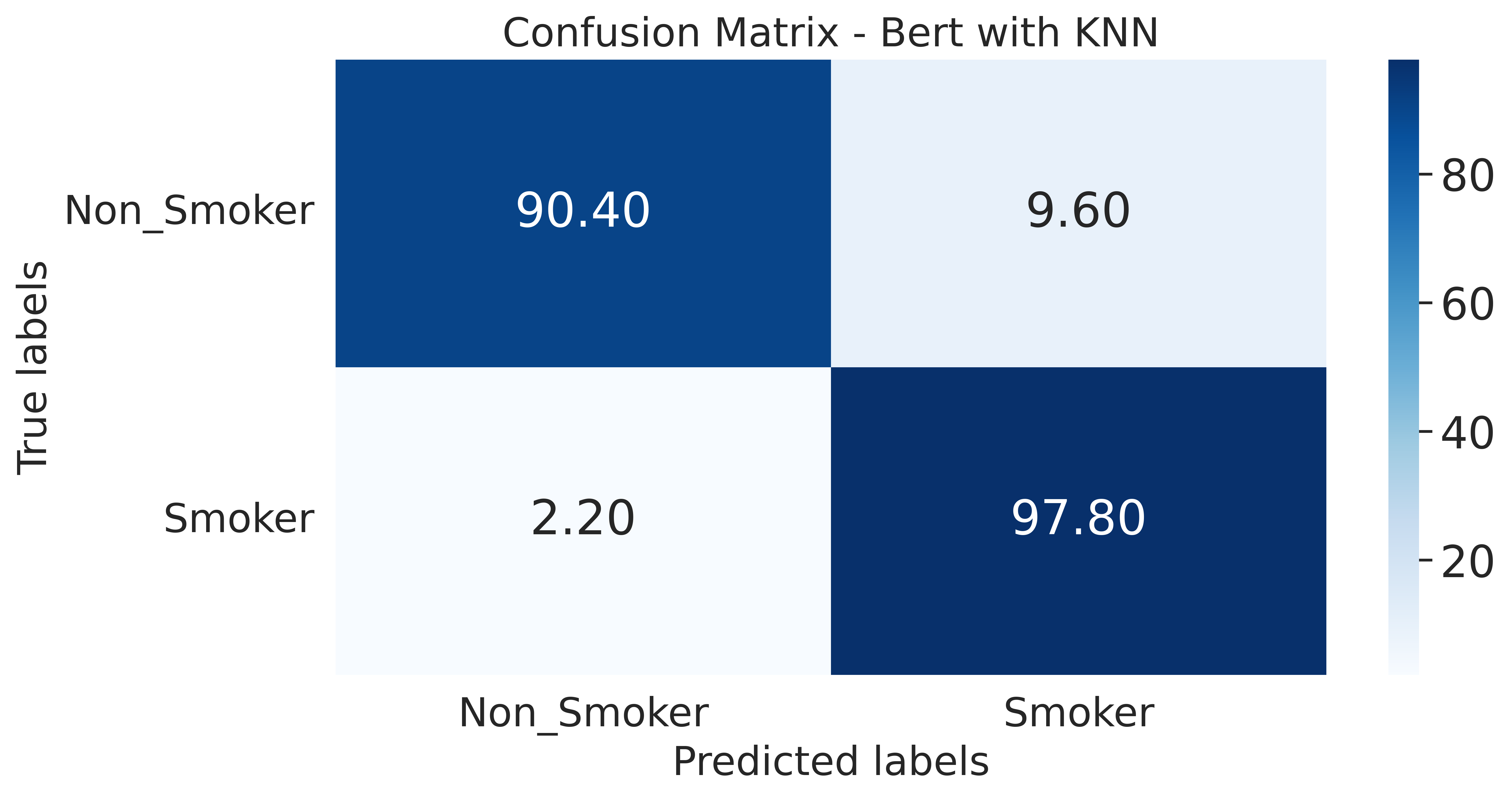 |
| 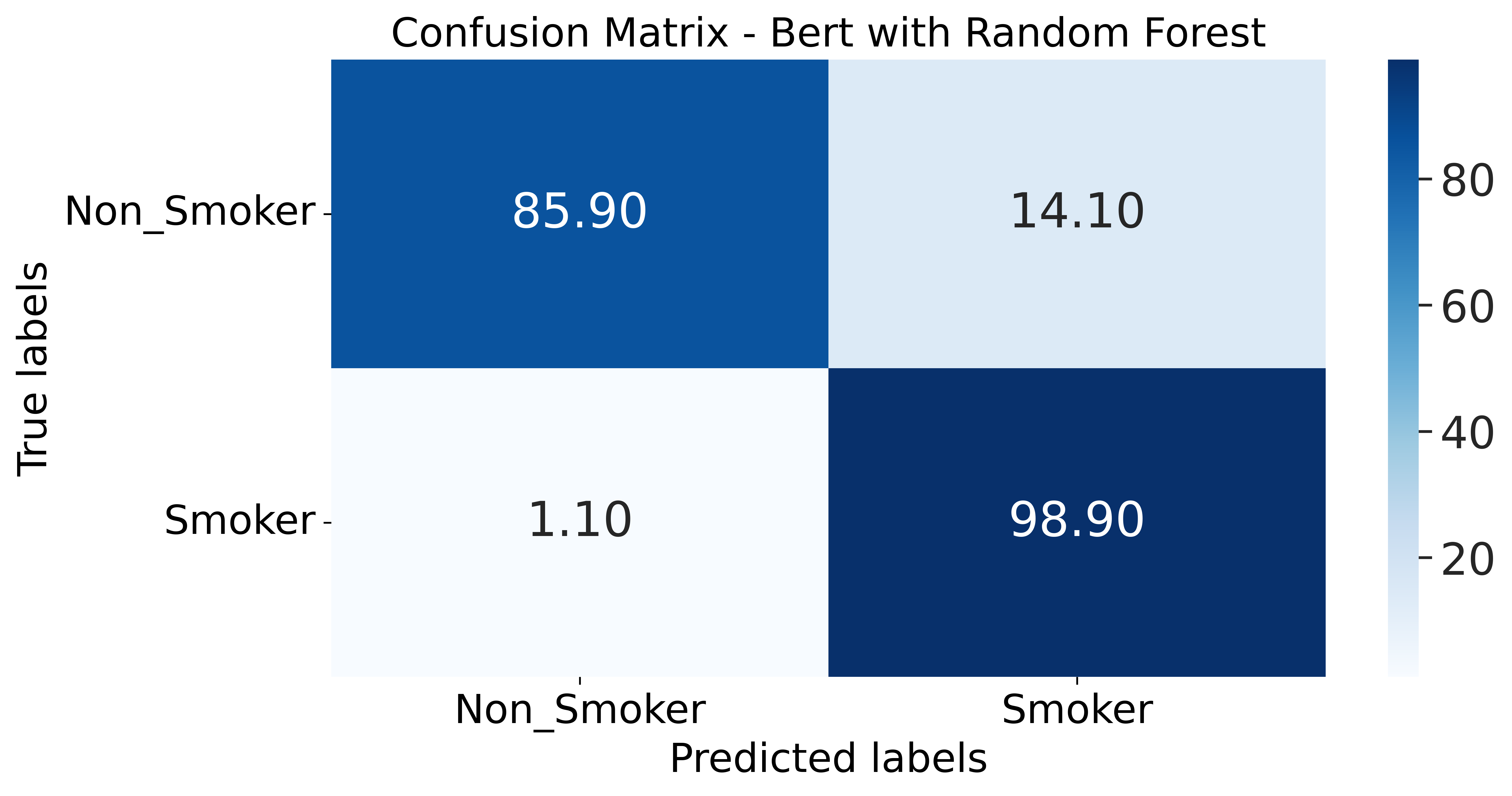 | 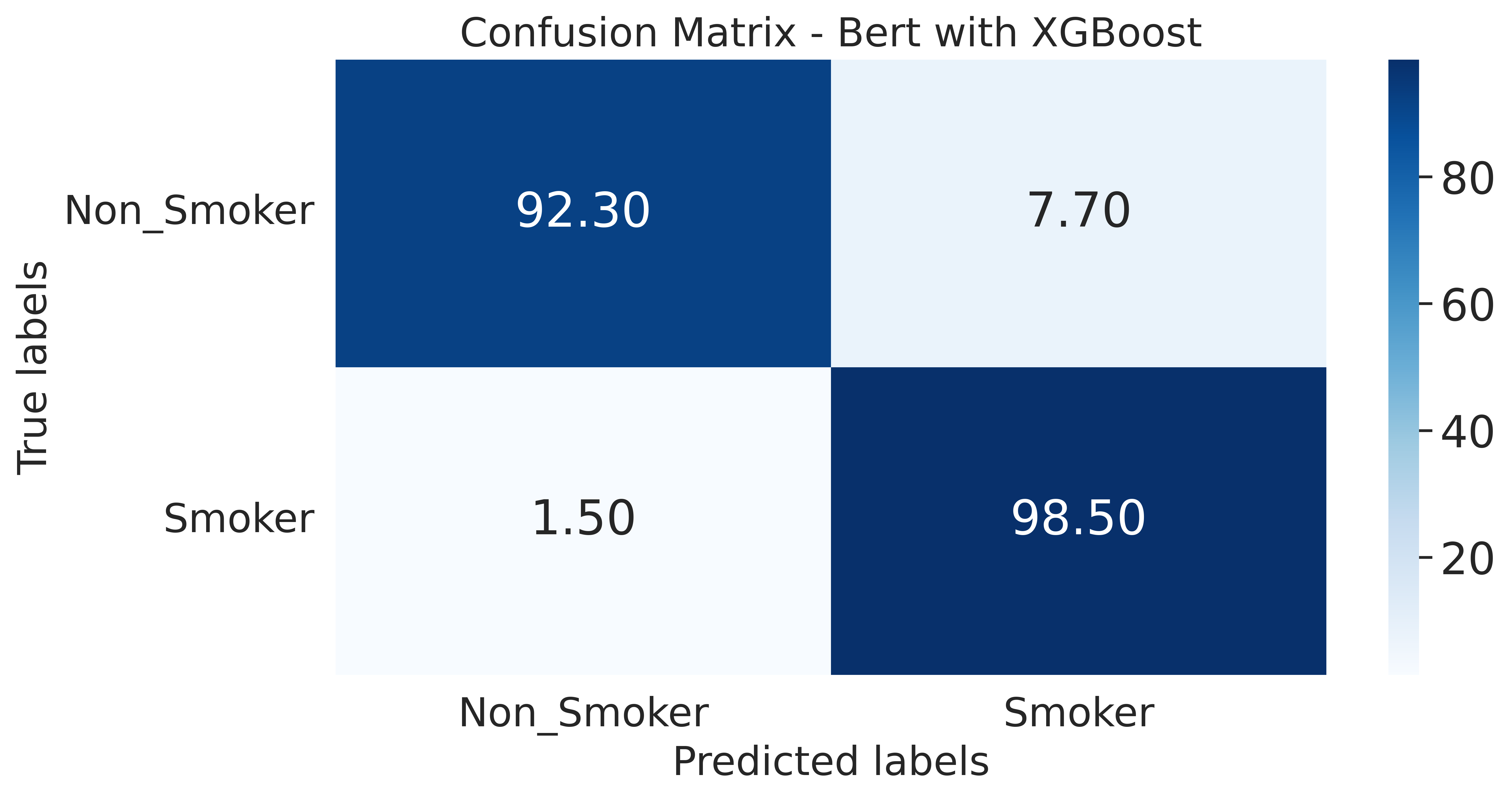 |
|  |  |
| Machine learning models with Count Vectorizer | |
| 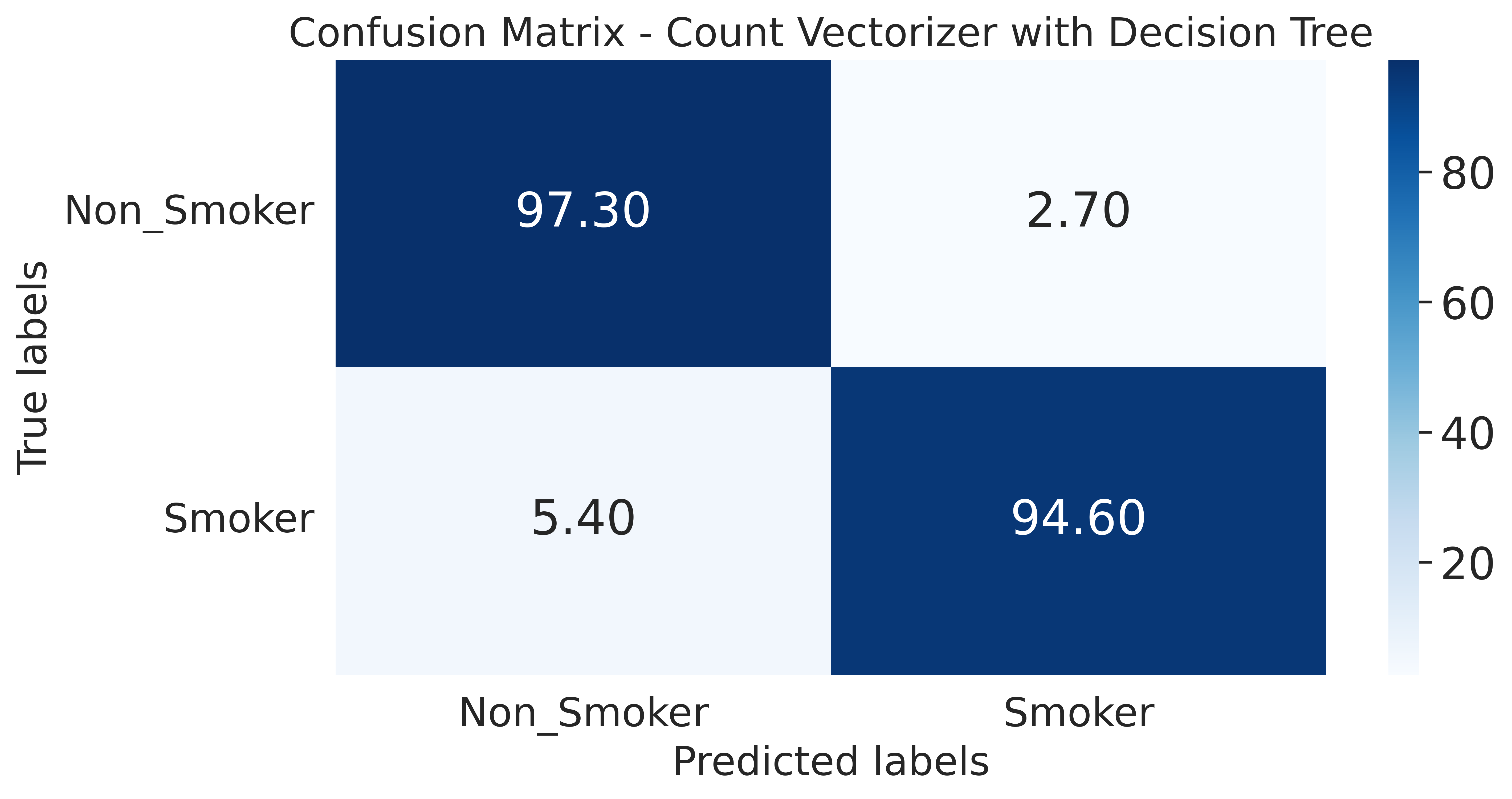 | 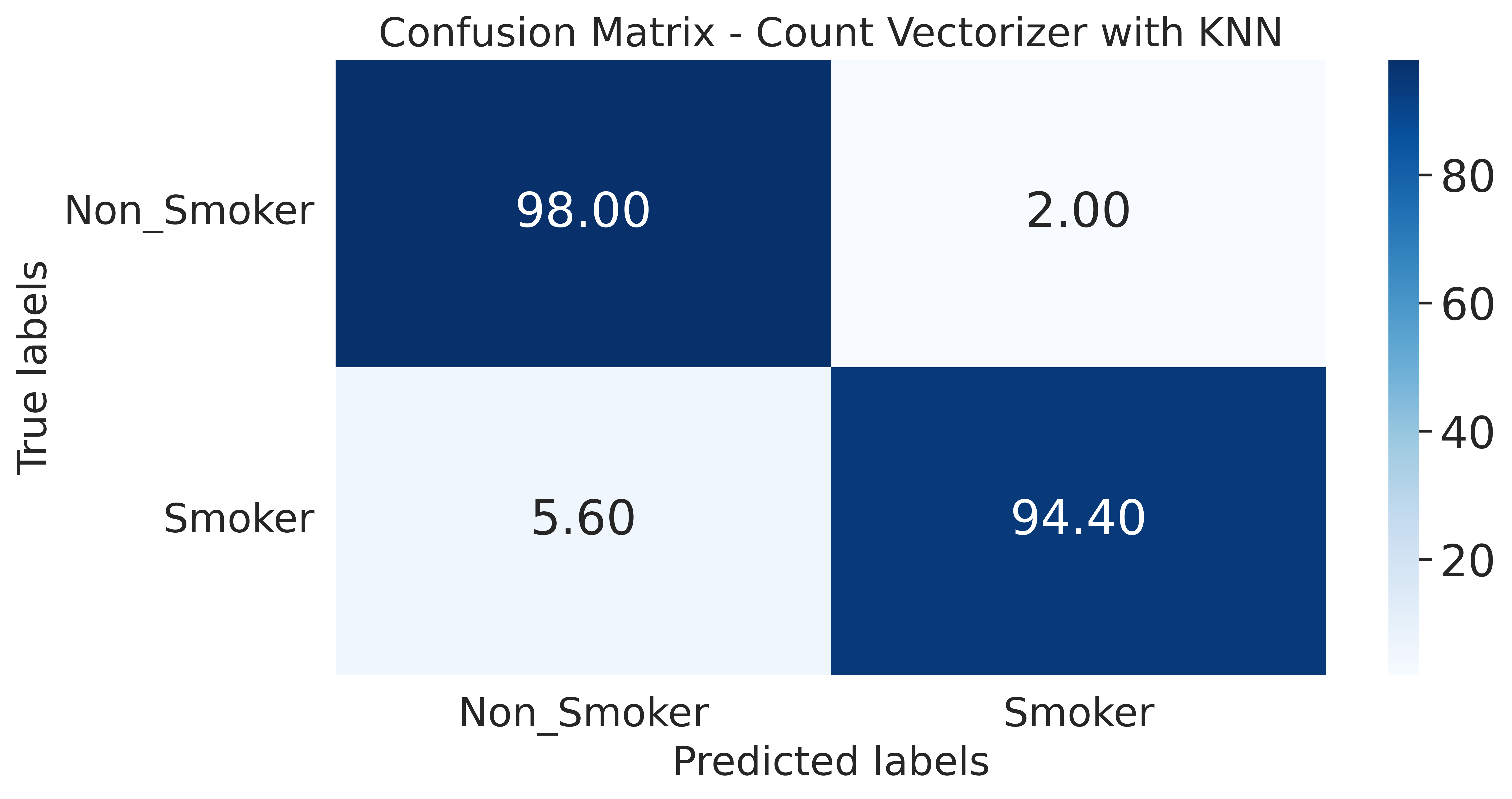 |
| 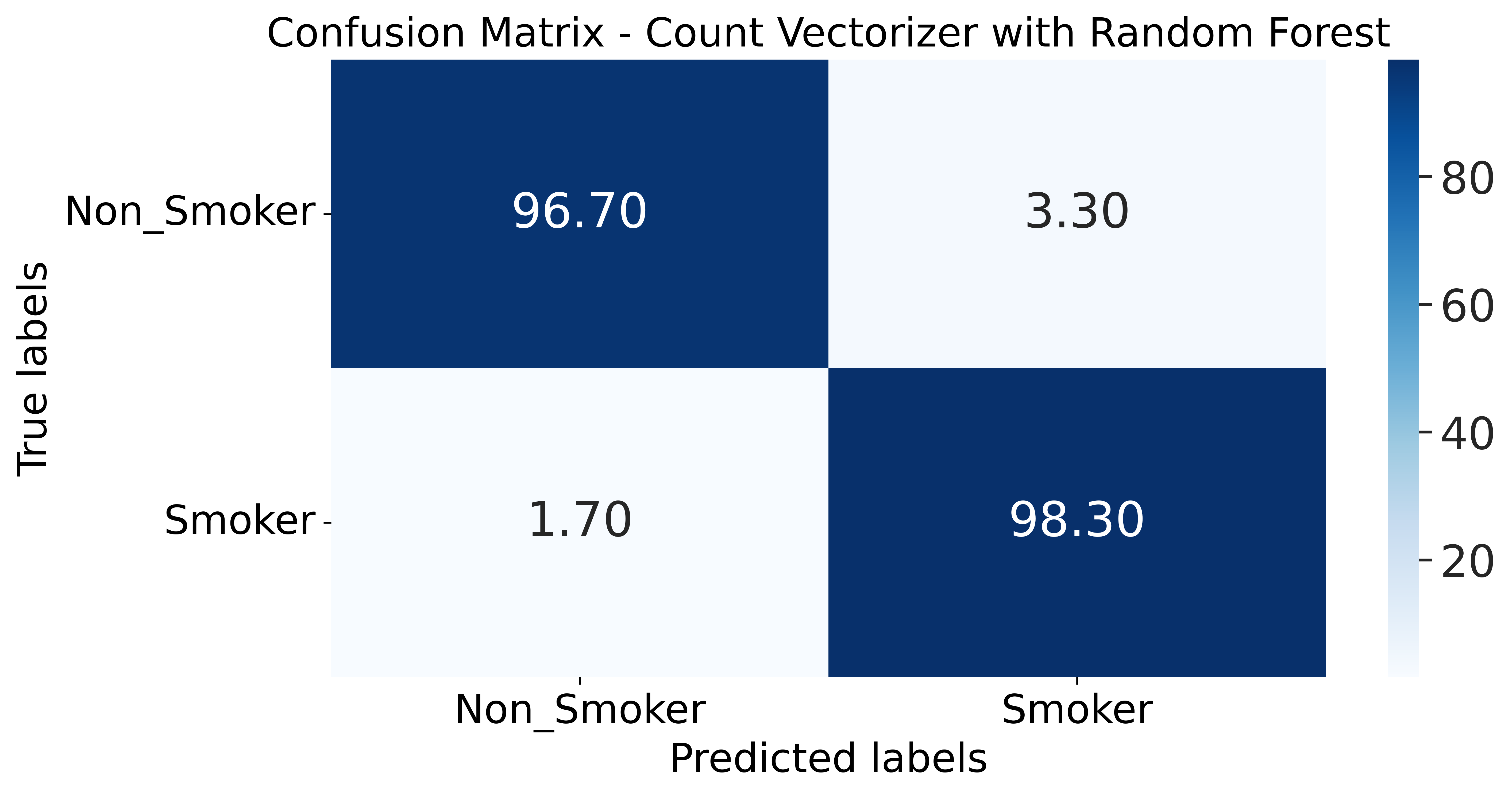 | 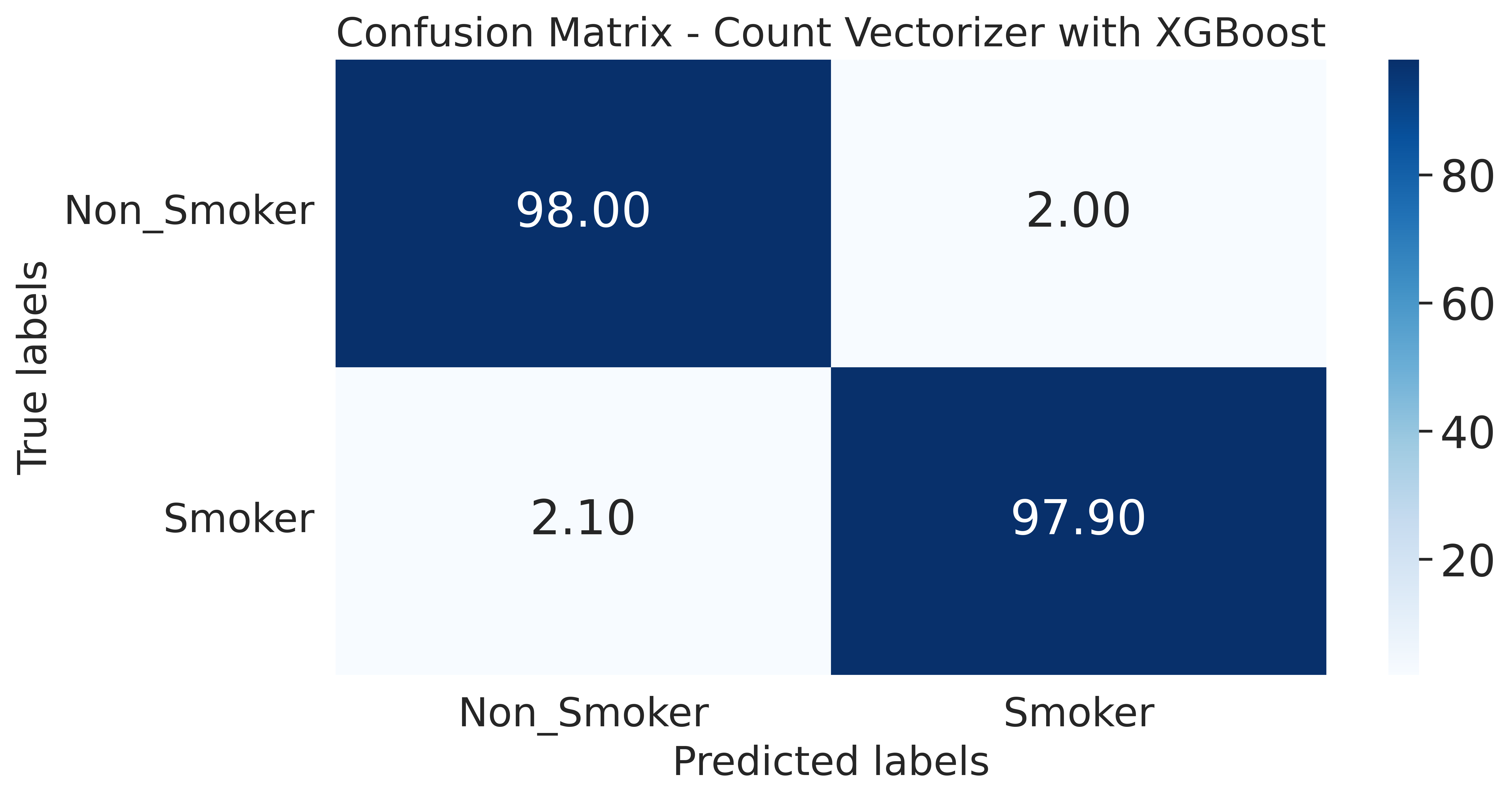 |
|  |  |

| **Table 1. Average performance measures based on binary classification of all developed models** | | | | | |
| --- | --- | --- | --- | --- | --- |
| **Word2Vector** | | | | | |
|  | Precision | Recall | F1-Score | Accuracy | ROC-AUC |
| KNN | 95% | 96% | 96% | 97% | 98% |
| DT | 95% | 96% | 96% | 96% | 96% |
| RF | 97% | 97% | 97% | 97% | 99% |
| XG-boost | 97% | 97% | 97% | 98% | 97% |
|  |  |  |  |  |  |
| **Embedding** | | | | | |
| KNN | 92% | 91% | 91% | 93% | 96% |
| DT | 95% | 95% | 95% | 96% | 96% |
| RF | 96% | 95% | 95% | 96% | 99% |
| XG-boost | 96% | 97% | 97% | 97% | 97% |
|  |  |  |  |  |  |
| **Bert** | | | | | |
| KNN | 95% | 94% | 95% | 96% | 96% |
| DT | 92% | 92% | 92% | 94% | 91% |
| RF | 96% | 92% | 94% | 95% | 99% |
| XG-boost | 96% | 95% | 96% | 97% | 95% |
|  |  |  |  |  |  |
| **Count Vectorizer** | | | | | |
| KNN | 93% | 96% | 94% | 95% | 98% |
| DT | 93% | 96% | 94% | 95% | 98% |
| RF | 97% | 97% | 97% | 98% | 99% |
| XG-boost | 97% | 98% | 97% | 98% | 98% |

# 2. Multiclass Classification

This section presents the results obtained from various machine learning classifiers: KNN, DT, RF, and XGBoost. These classifiers were developed utilizing features extracted through different feature extraction methods to classify samples into Never-Smoker, Former-Smoker and 'Active-Smoker' classes.

| Figure 2. Confusion matrixes based on multiclass classification of all developed models. | |
| --- | --- |
| Machine learning models with Word2Vector | |
| 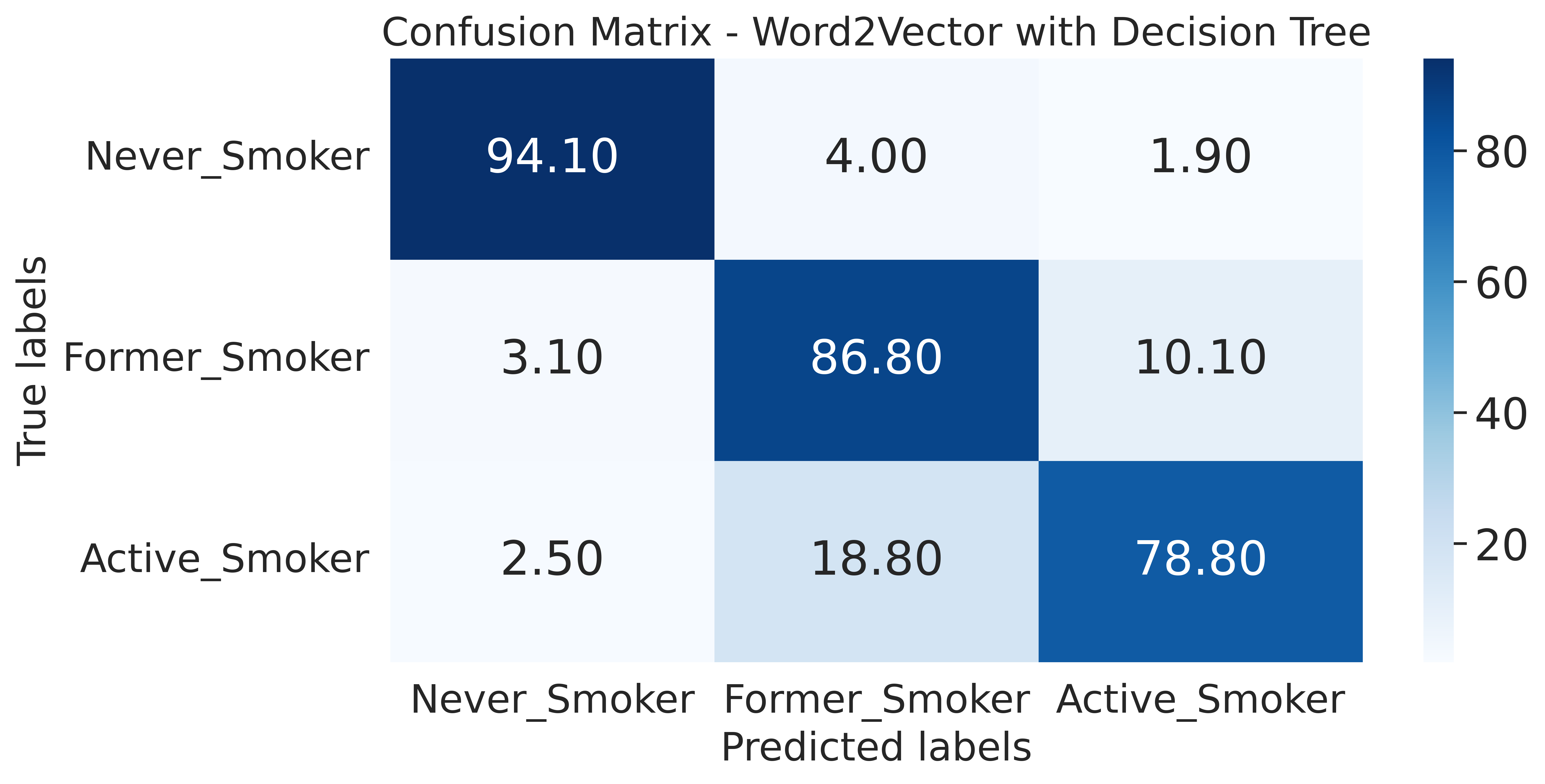 | 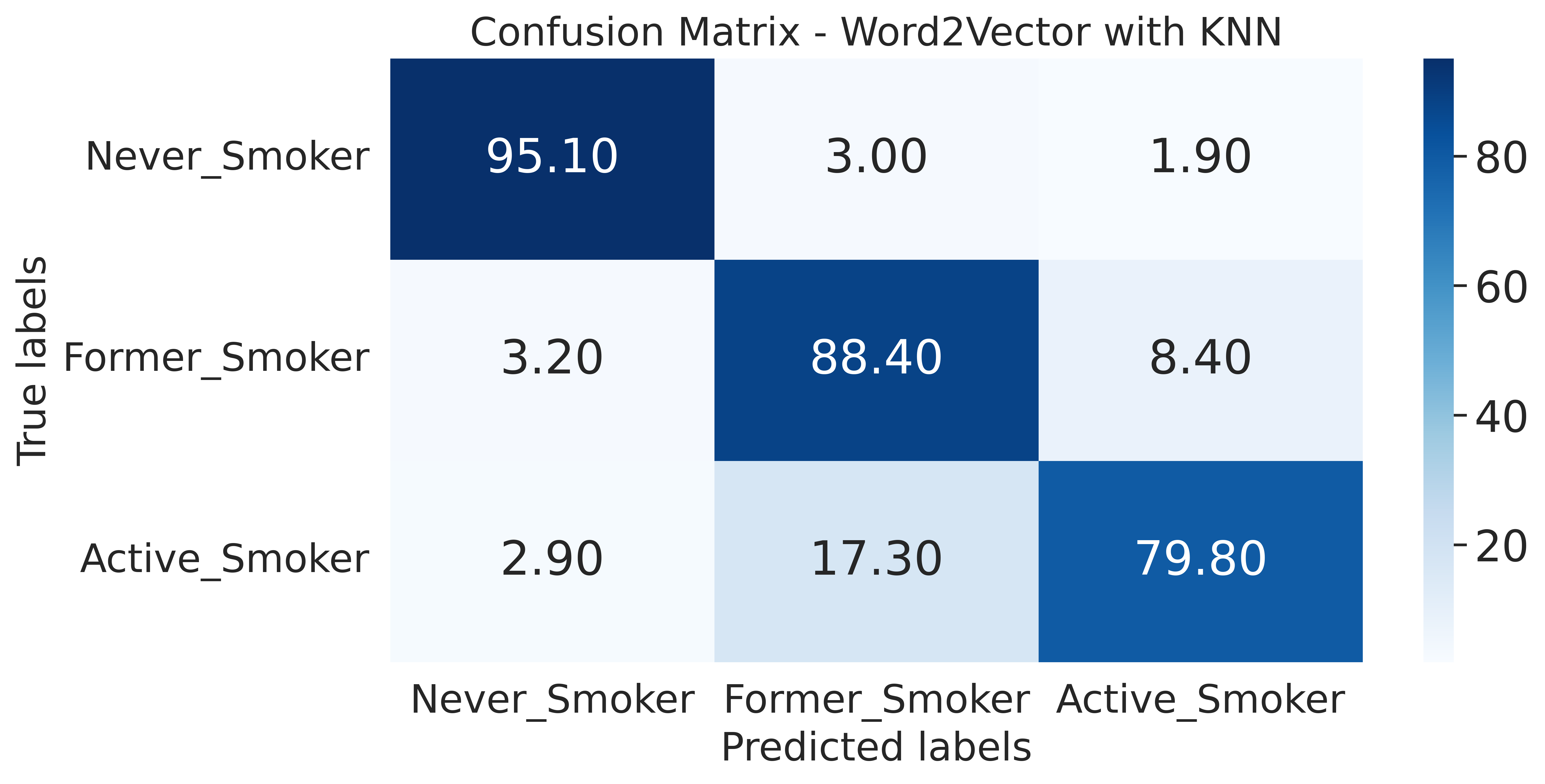 |
| 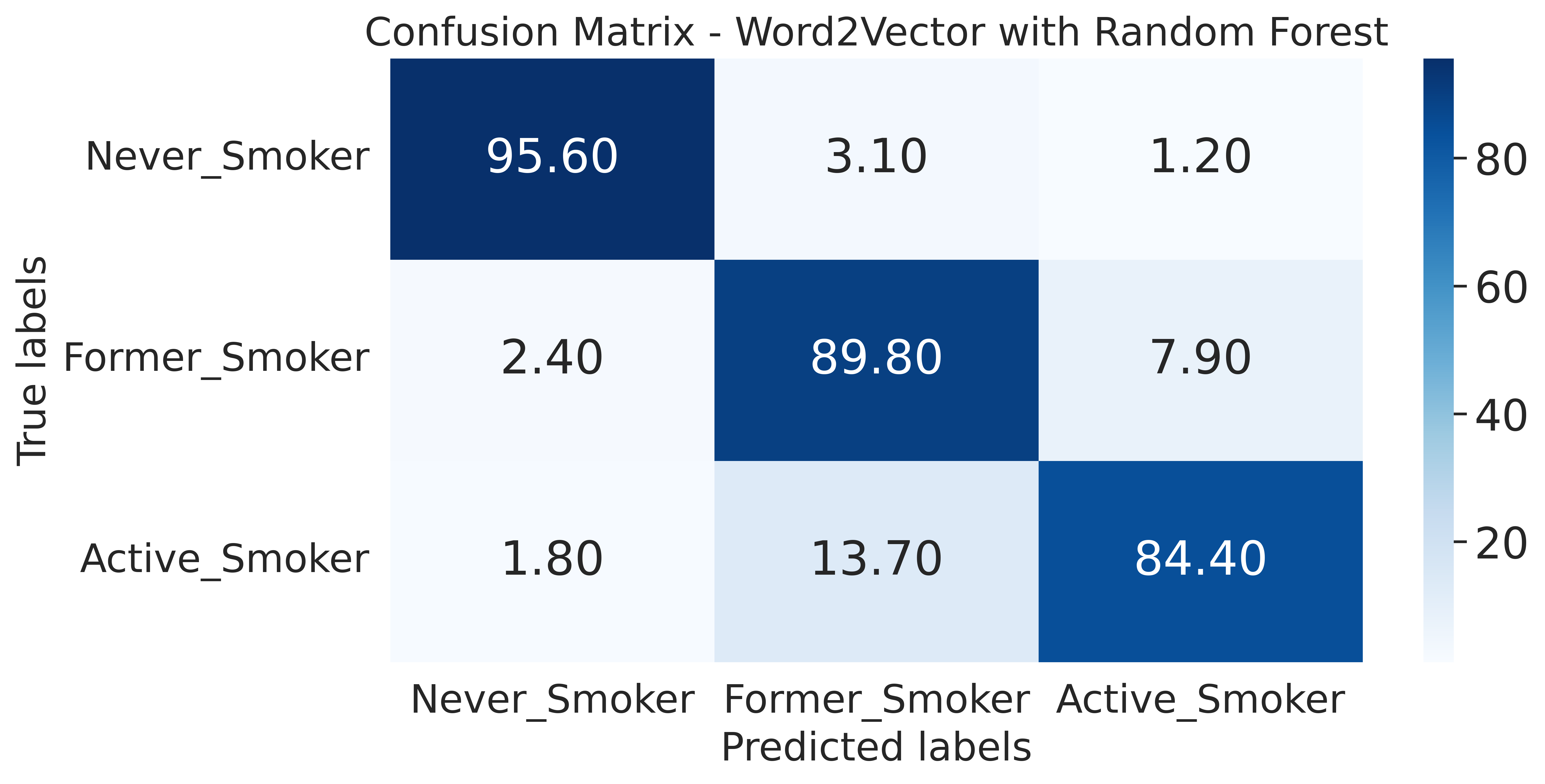 | 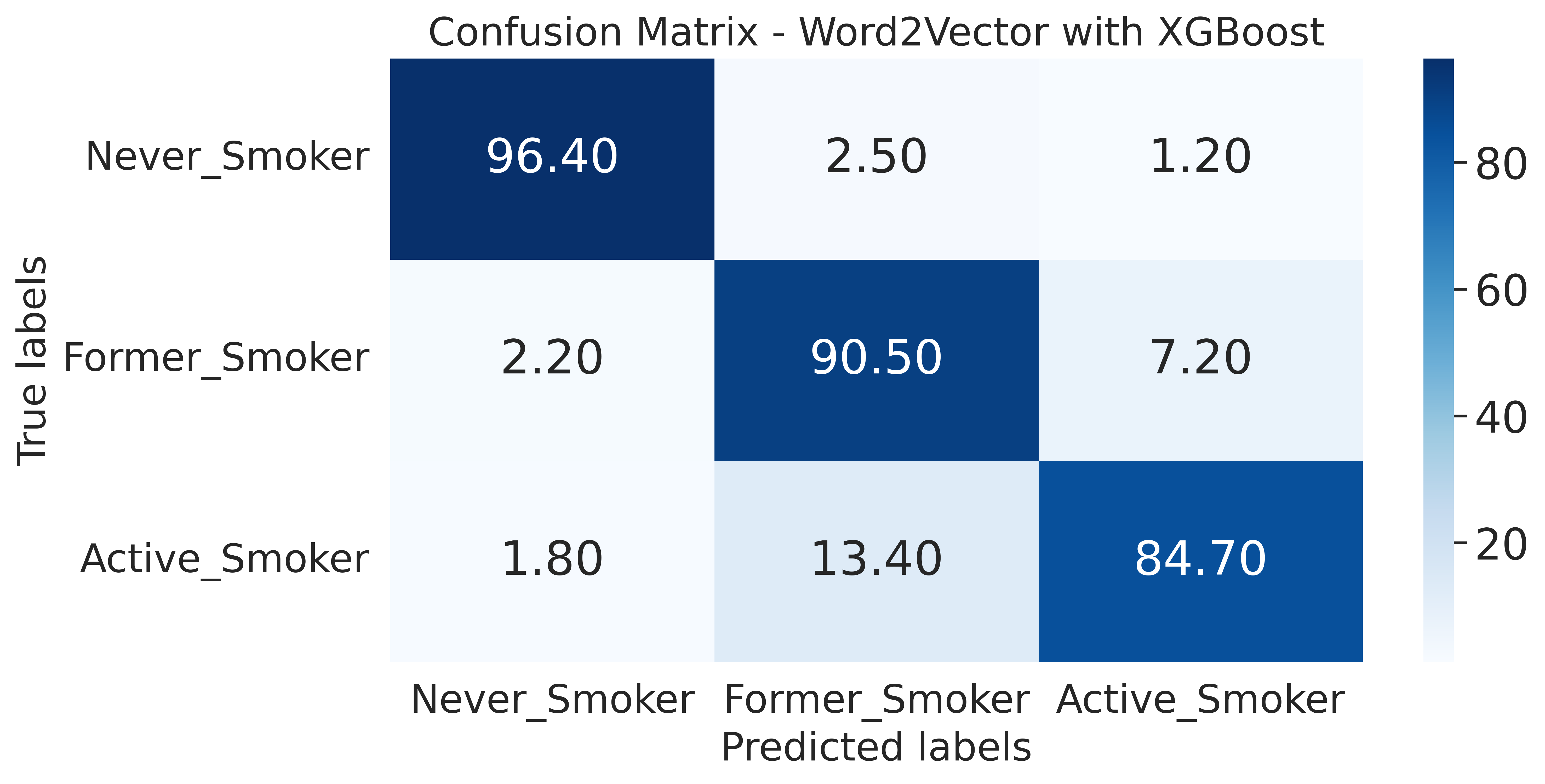 |
|  |  |
| Machine learning models with Embedding | |
| 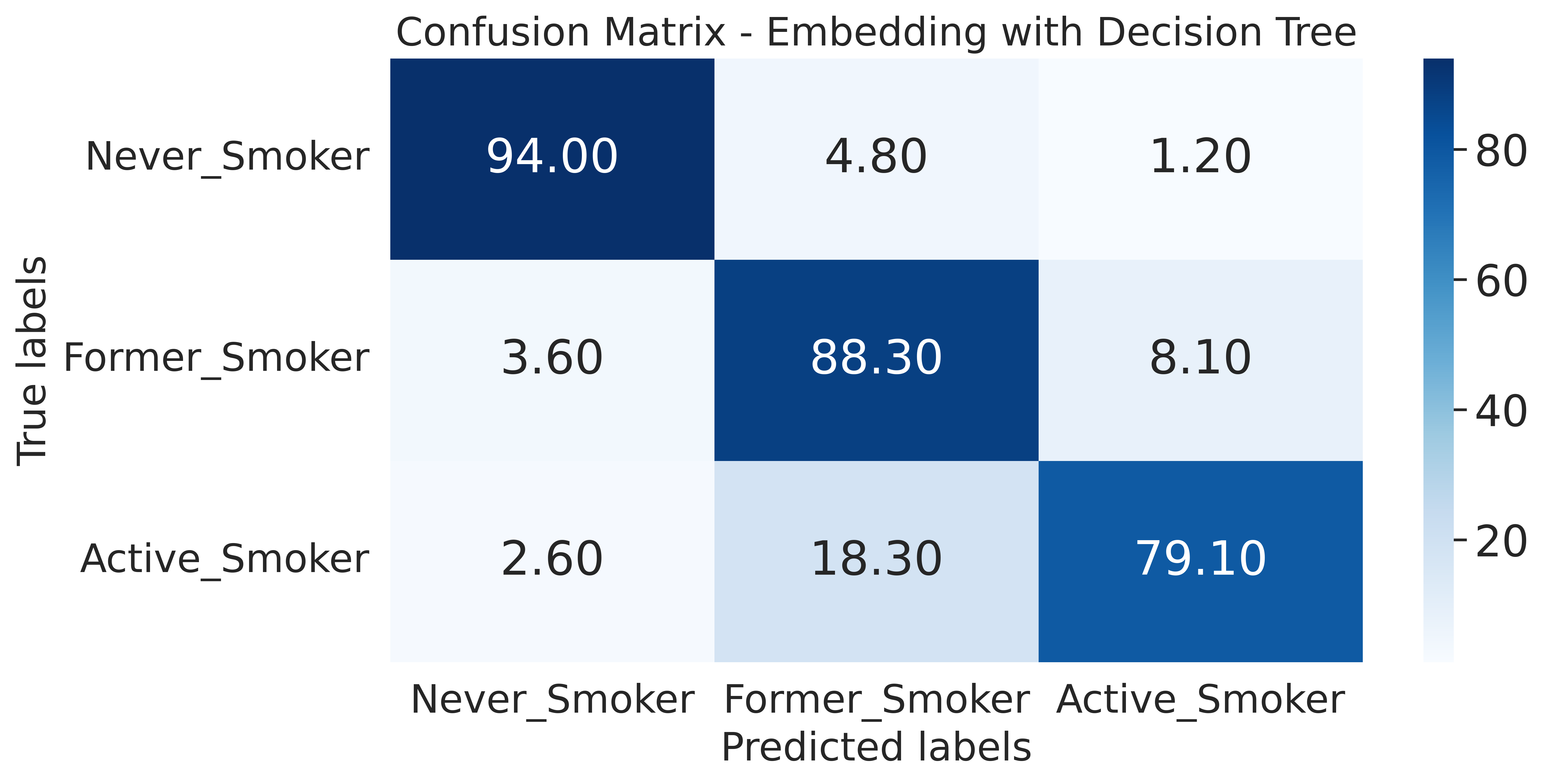 | 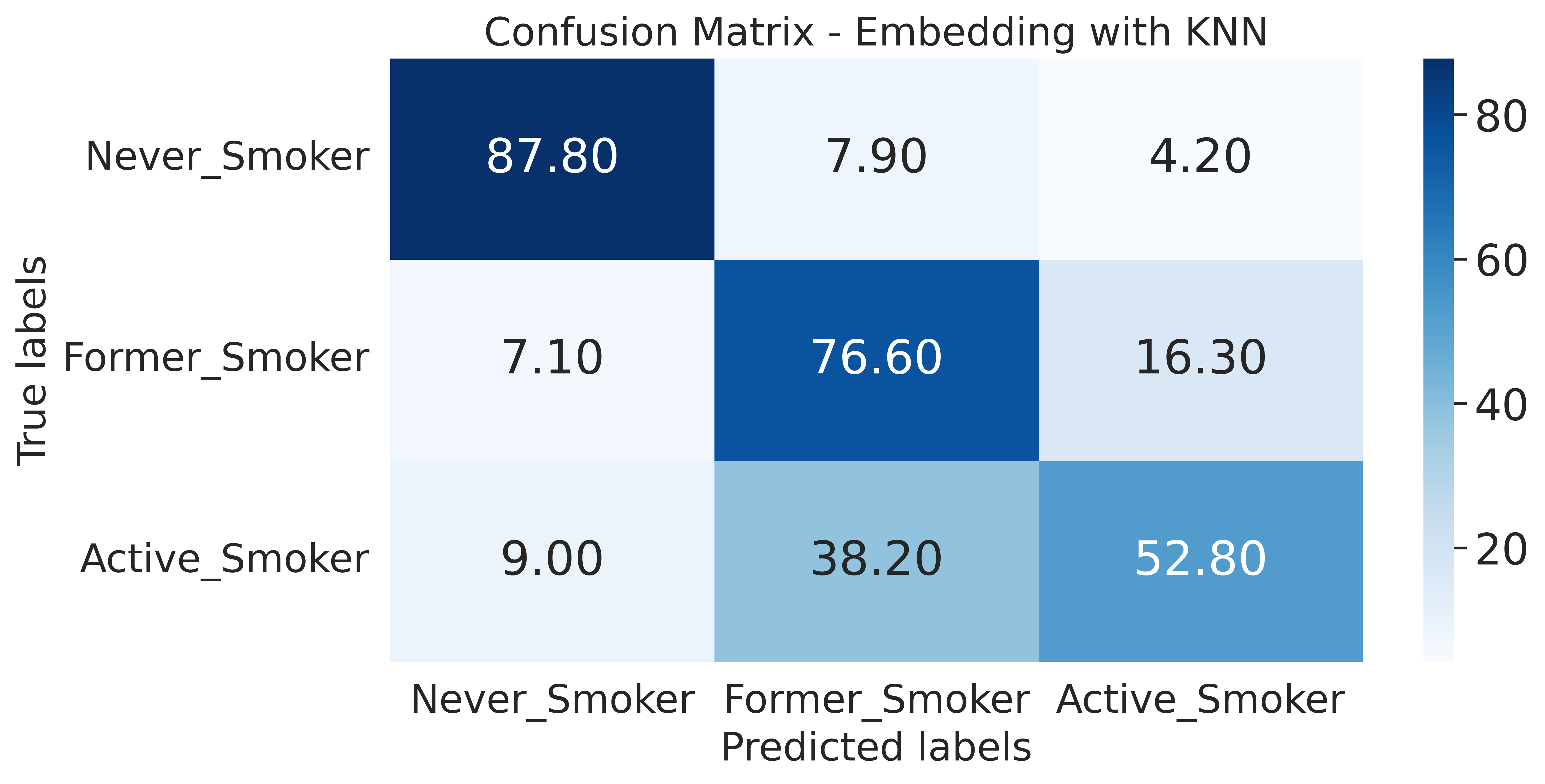 |
| 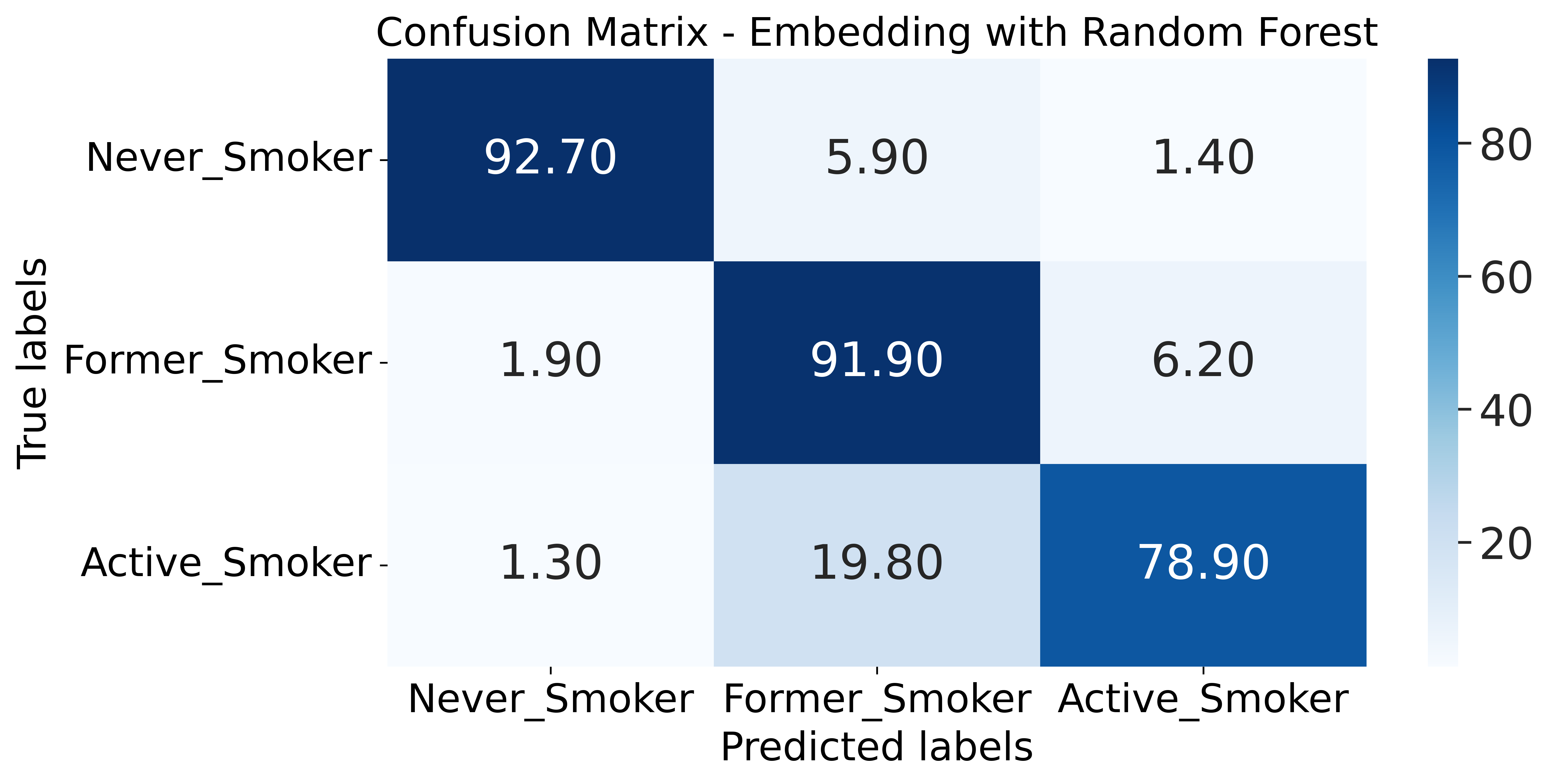 | 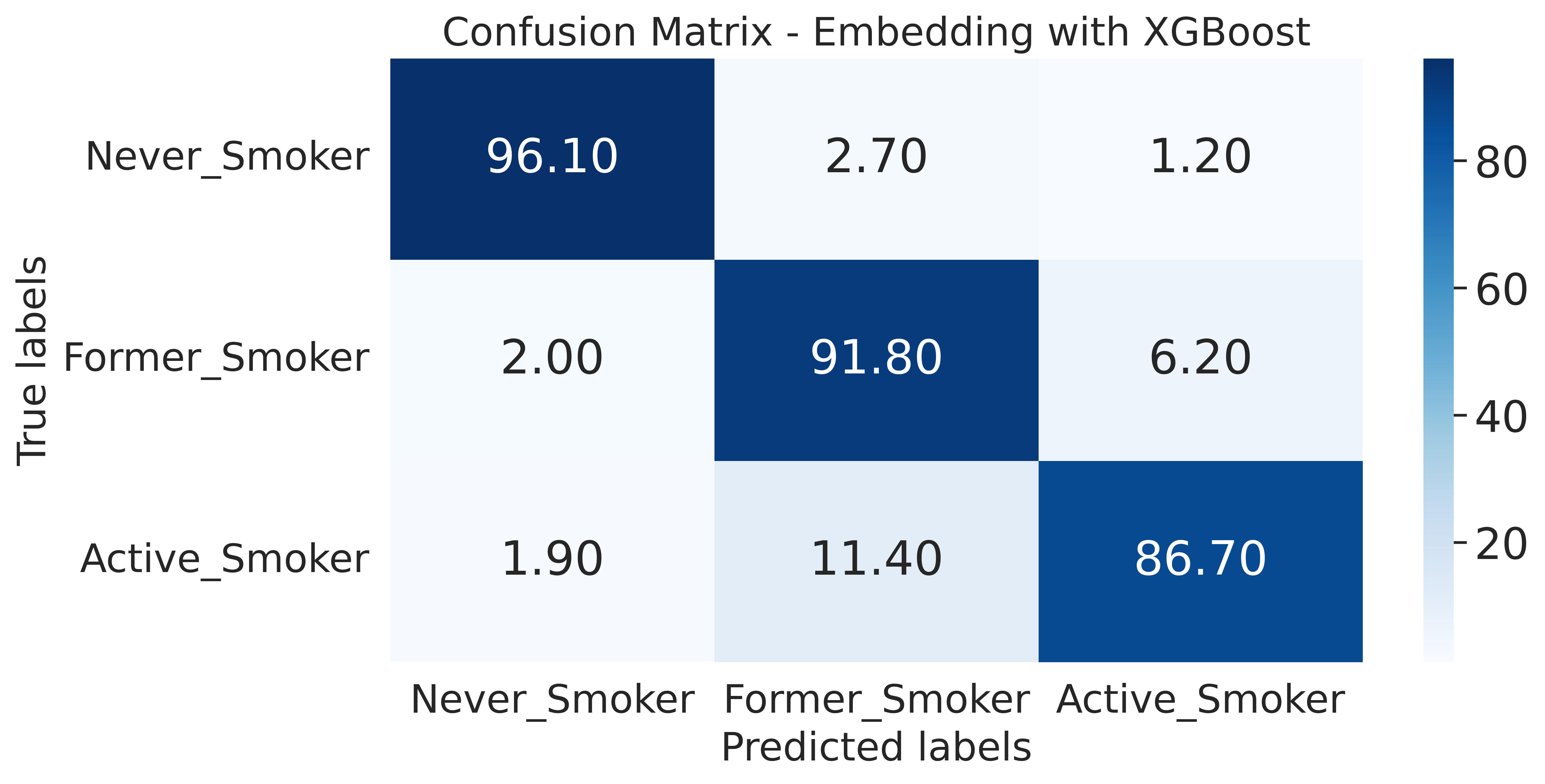 |
|  |  |
| Machine learning models with Bert | |
| 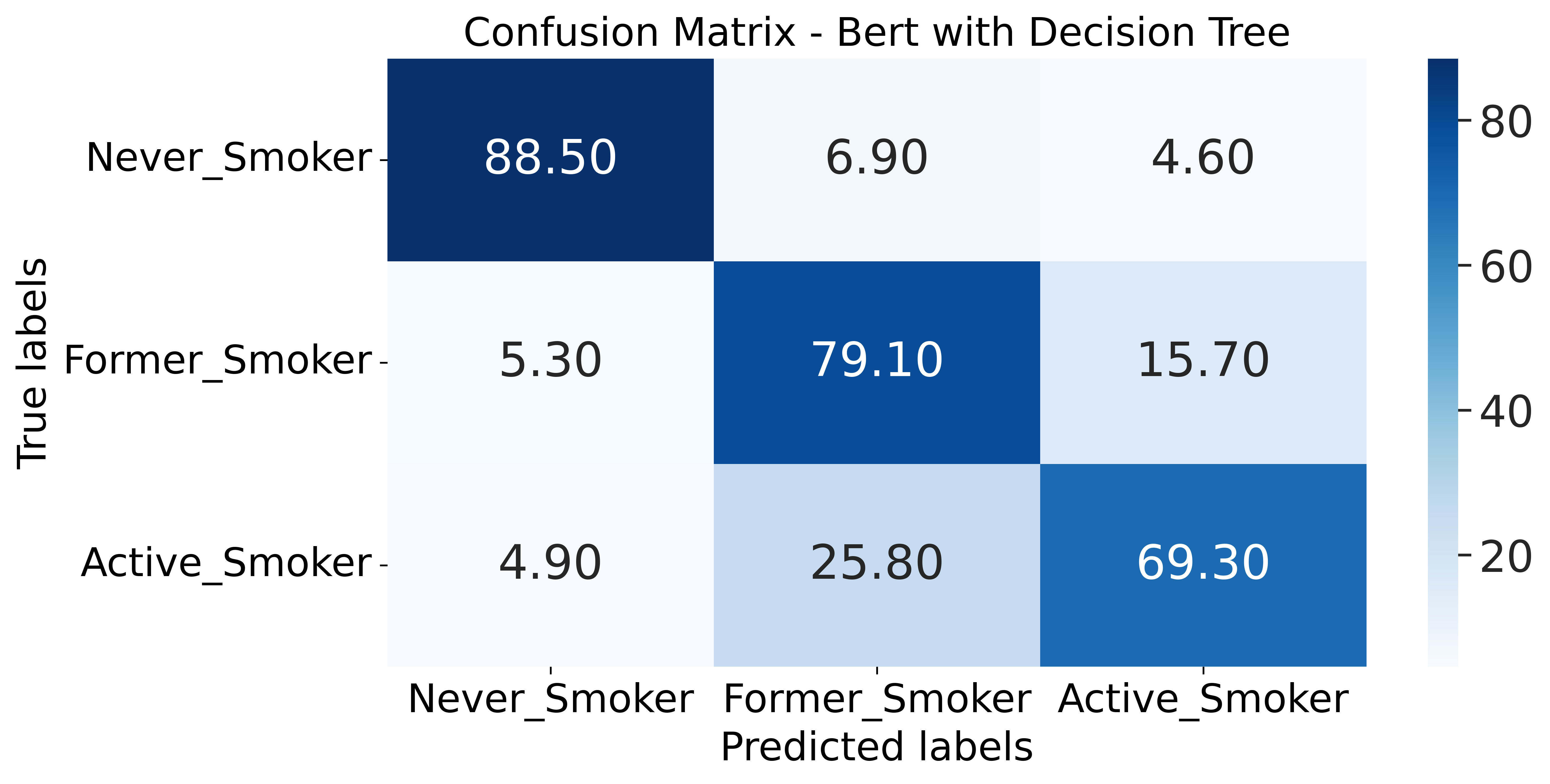 | 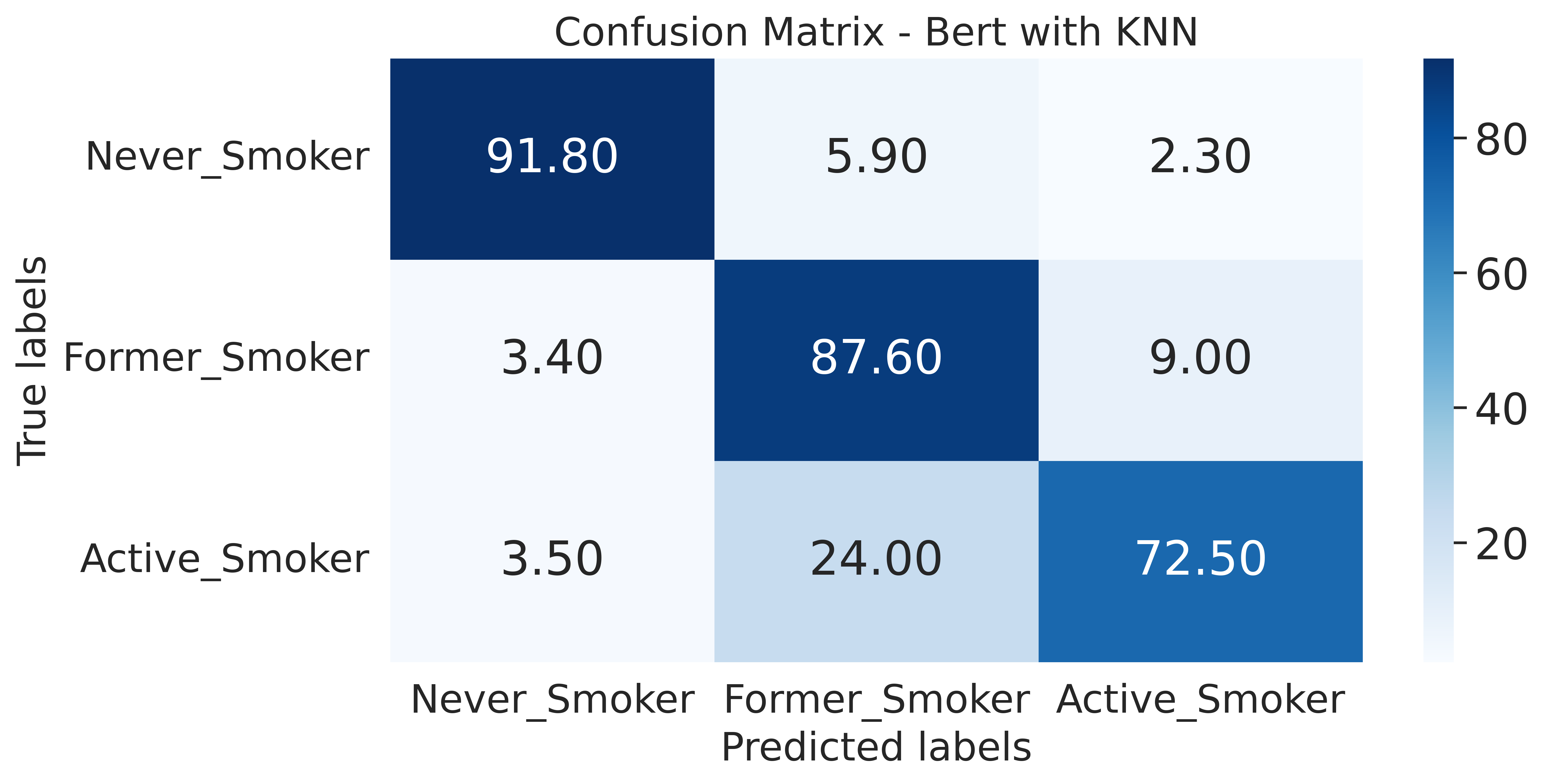 |
| 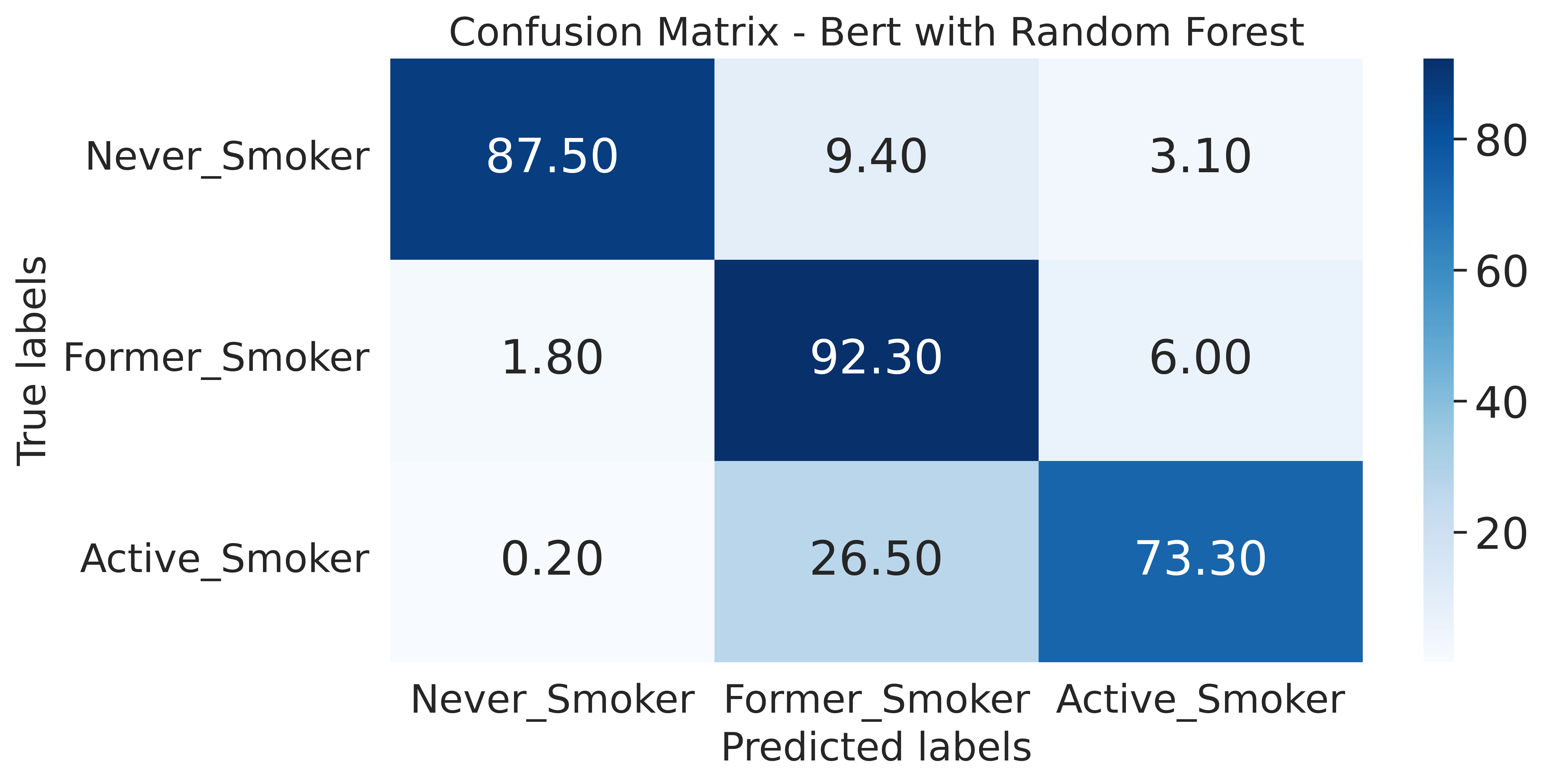 | 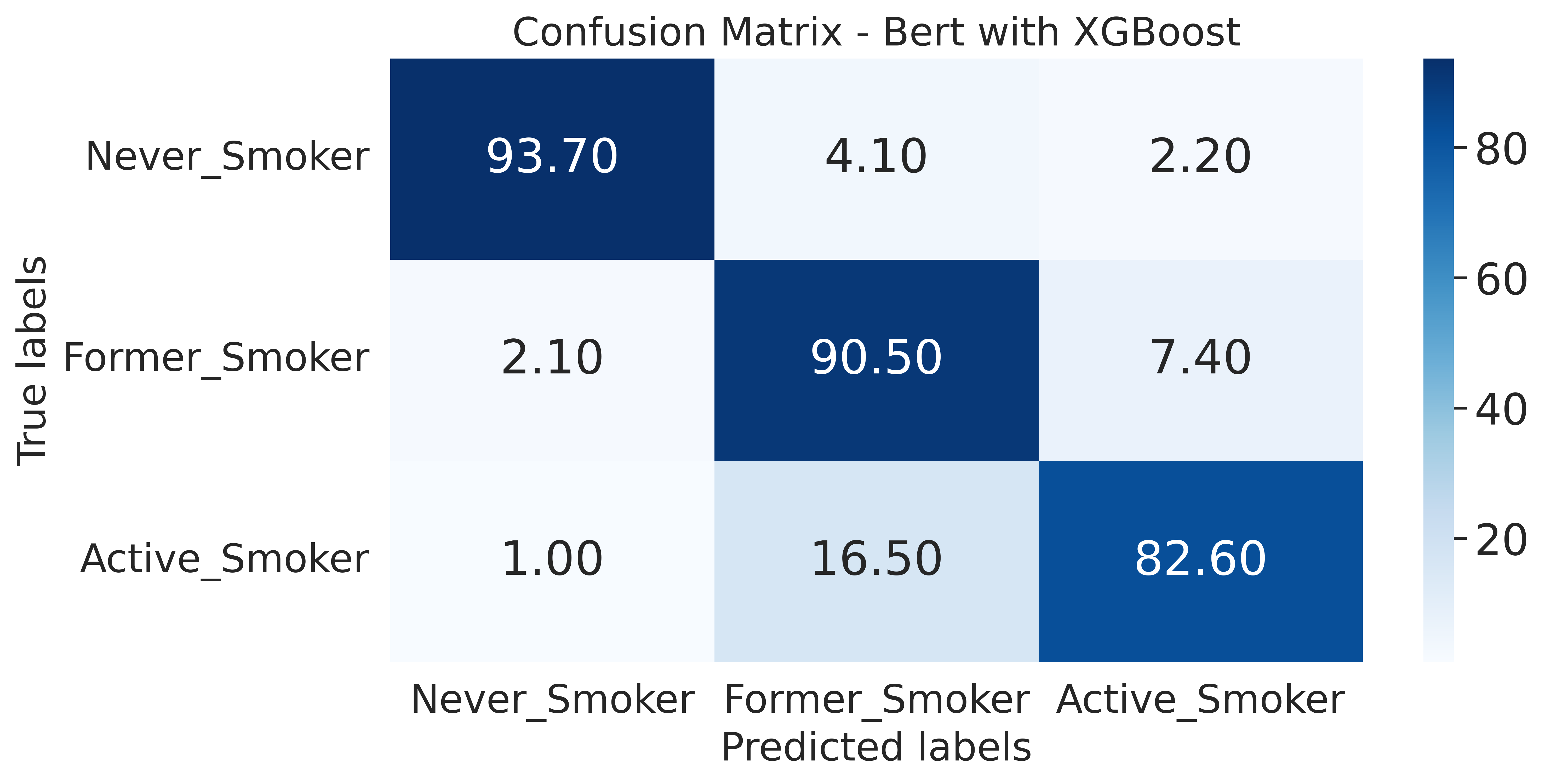 |
|  |  |
| Machine learning models with Count Vectorizer | |
| 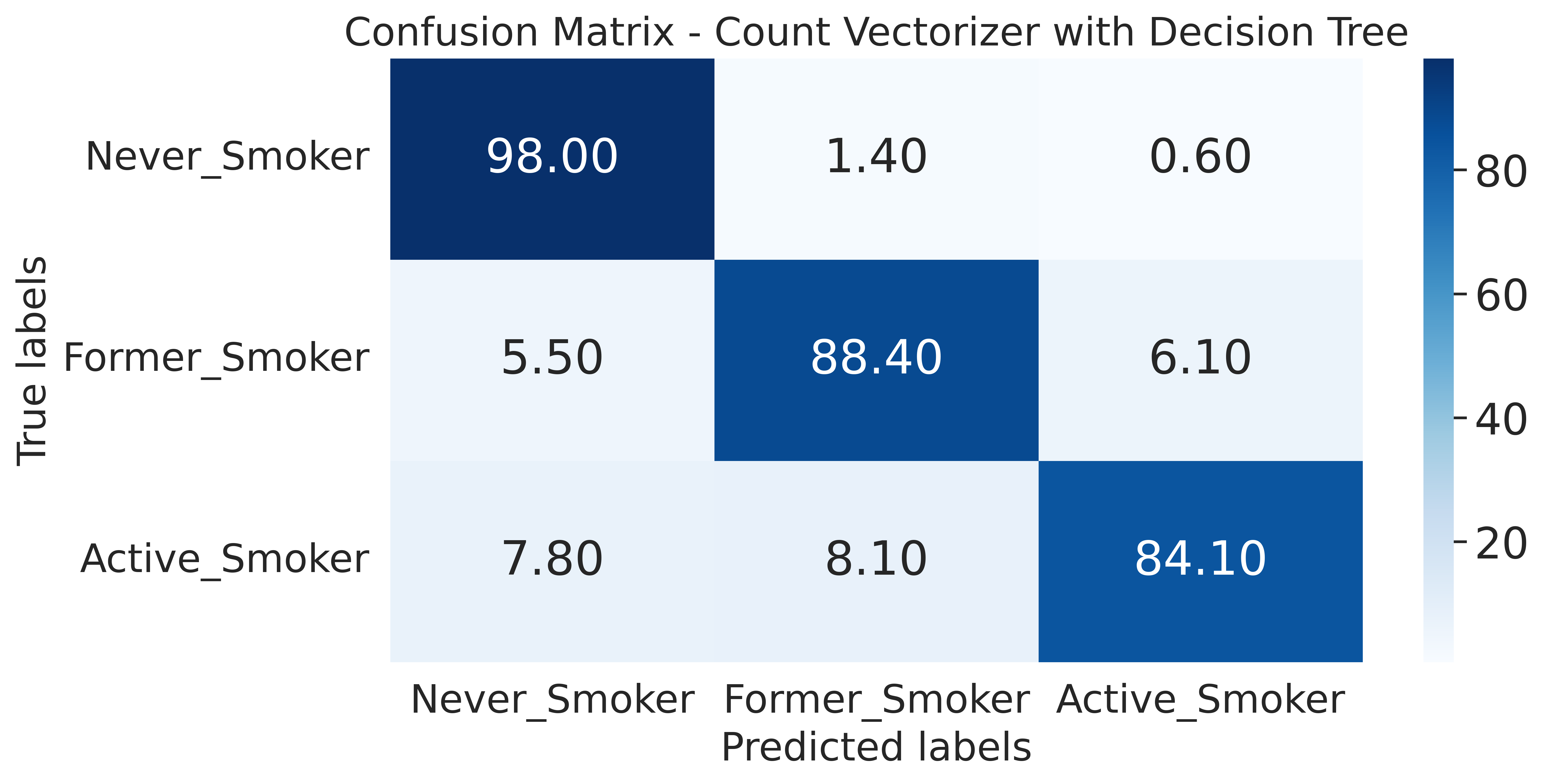 | 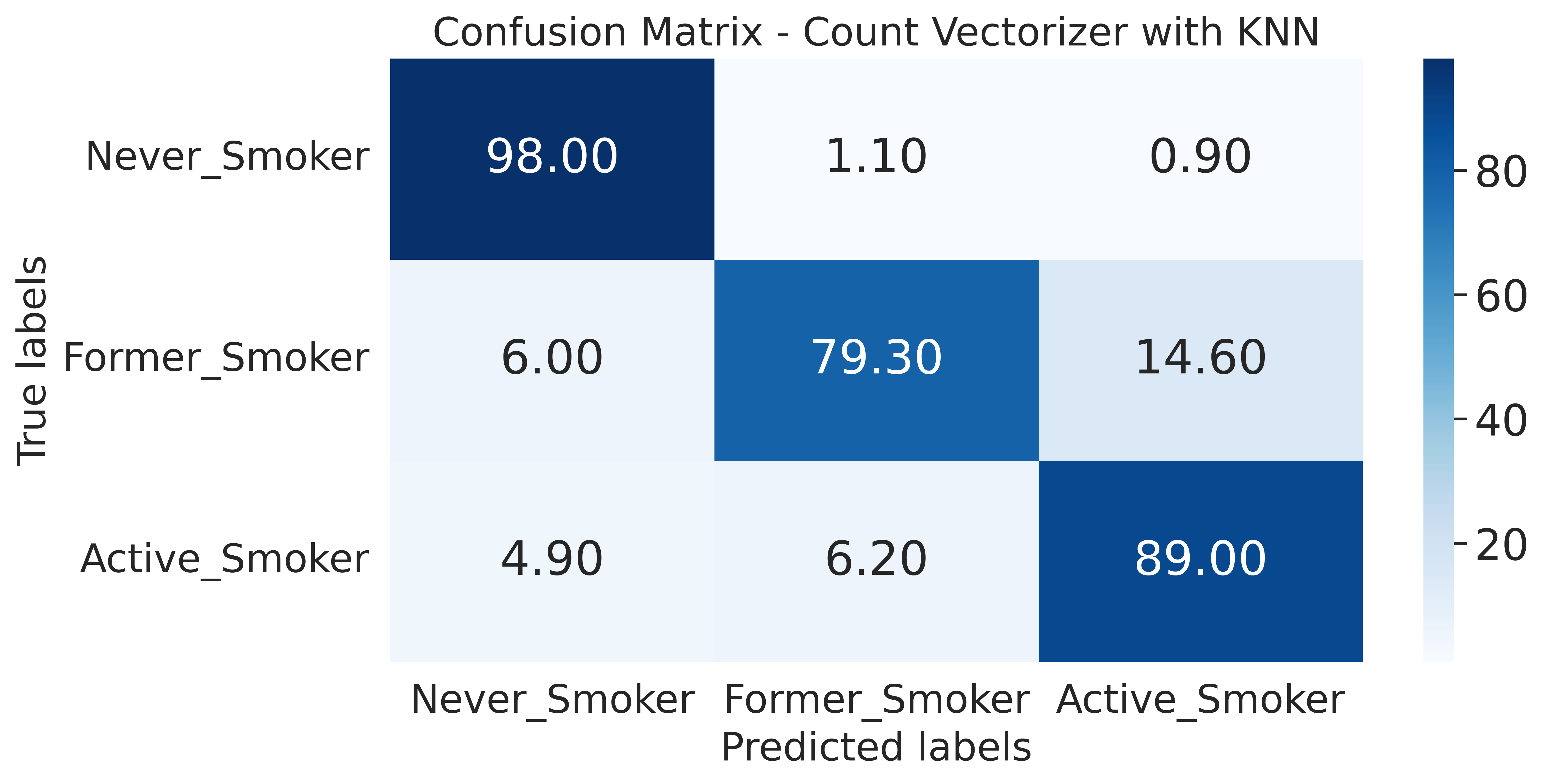 |
| 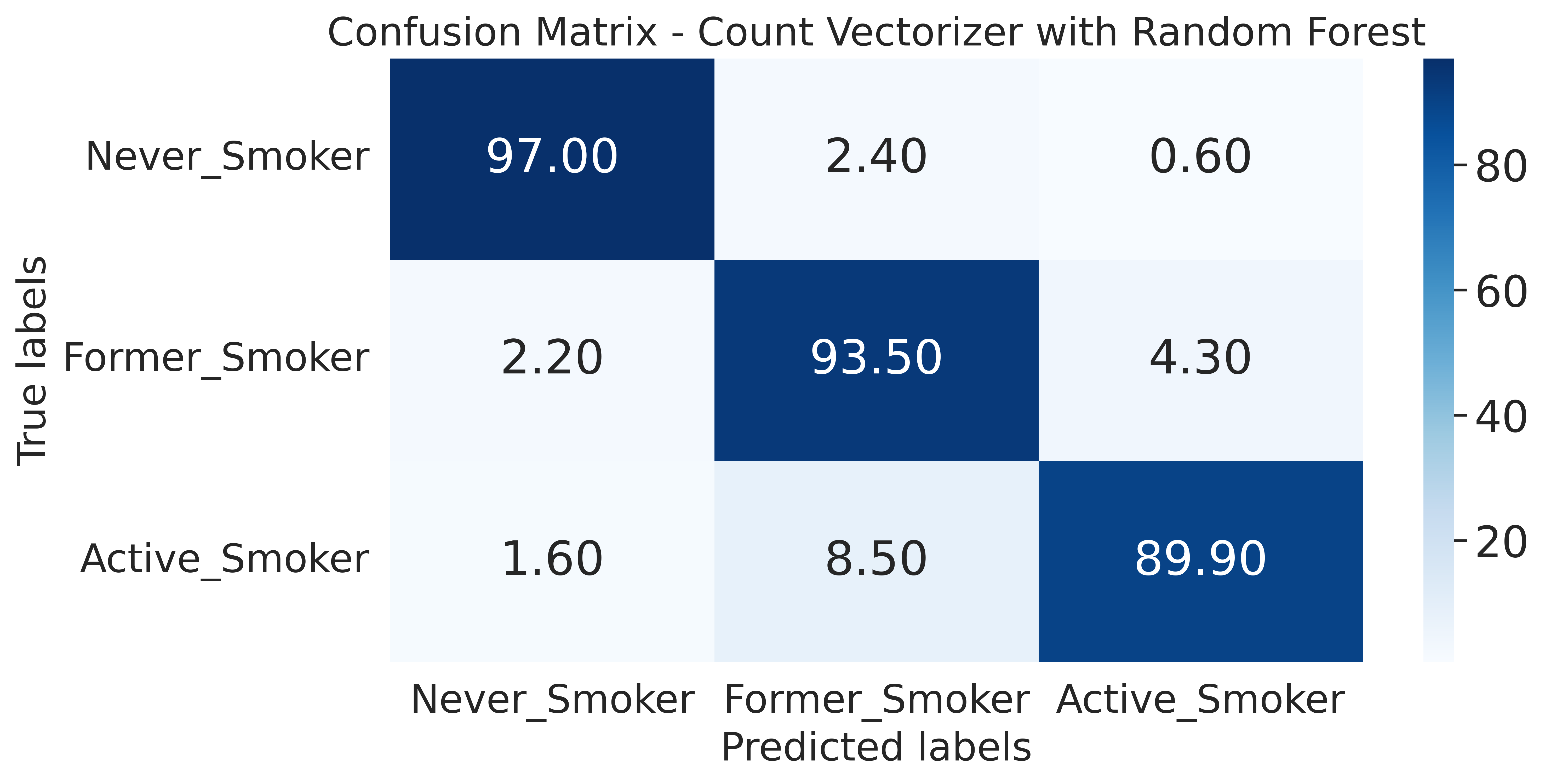 | 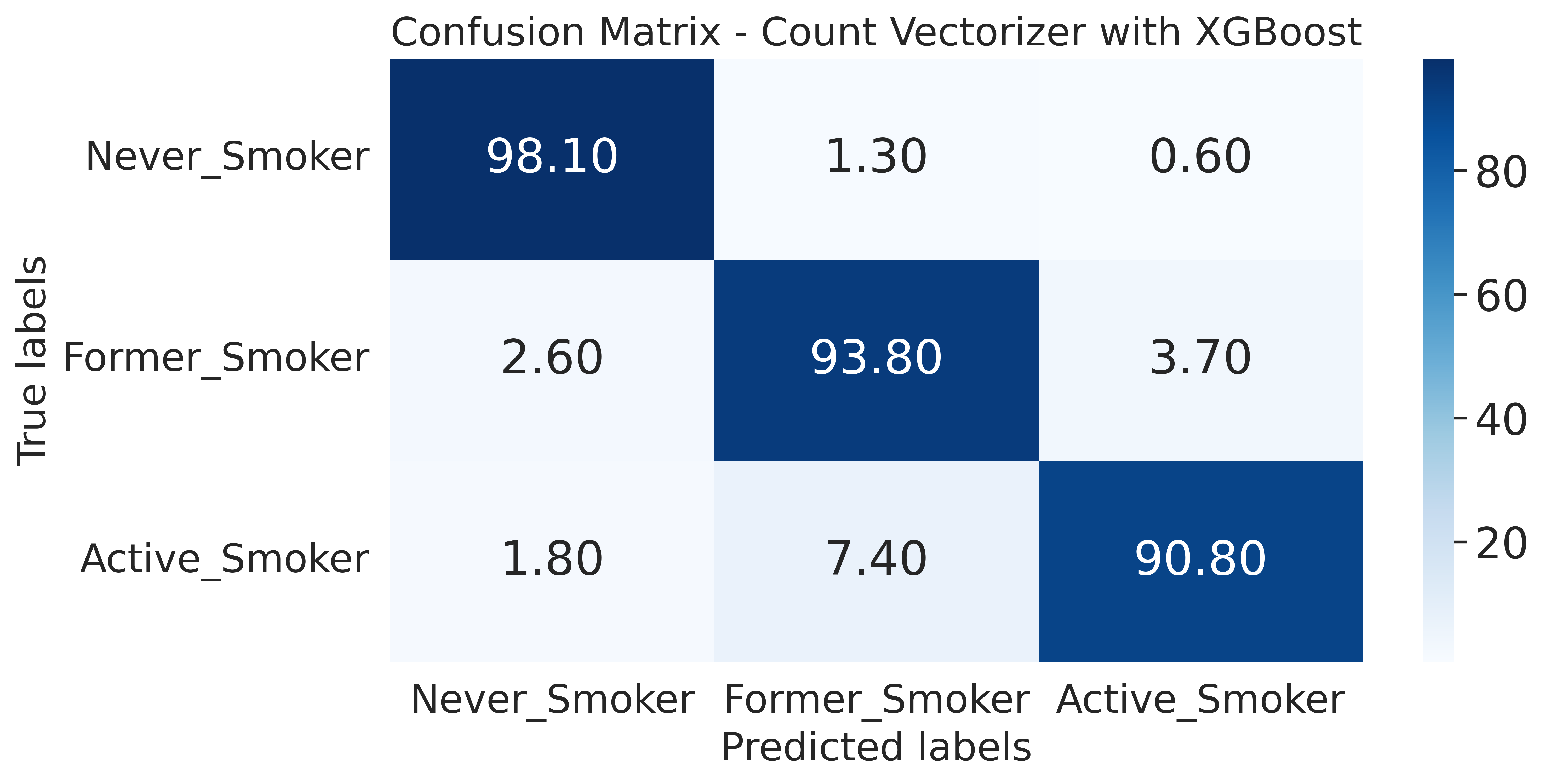 |
|  |  |

| **Table 2. Average performance measures based on multiclass classification of all developed models** | | | | | |
| --- | --- | --- | --- | --- | --- |
| **Word2Vector** | | | | | |
|  | Precision | Recall | F1-Score | Accuracy | ROC-AUC |
| KNN | 88% | 88% | 88% | 88% | 95% |
| DT | 86% | 86% | 86% | 86% | 89% |
| RF | 90% | 90% | 90% | 90% | 98% |
| XG-boost | 91% | 91% | 91% | 91% | 97% |
|  |  |  |  |  |  |
| **Embedding** | | | | | |
| KNN | 72% | 72% | 72% | 73% | 88% |
| DT | 87% | 87% | 87% | 87% | 94% |
| RF | 89% | 88% | 88% | 88% | 98% |
| XG-boost | 92% | 92% | 92% | 92% | 98% |
|  |  |  |  |  |  |
| **Bert** | | | | | |
| KNN | 85% | 84% | 84% | 84% | 93% |
| DT | 79% | 79% | 79% | 79% | 83% |
| RF | 88% | 84% | 86% | 86% | 97% |
| XG-boost | 90% | 89% | 89% | 89% | 98% |
|  | 85% | 84% | 84% | 84% | 93% |
| **Count Vectorizer** | | | | | |
| KNN | 86% | 89% | 87% | 87% | 93% |
| DT | 89% | 90% | 89% | 90% | 96% |
| RF | 93% | 93% | 93% | 93% | 99% |
| XG-boost | 94% | 94% | 94% | 94% | 99% |
